# Advancing understanding of long COVID pathophysiology through quantum walk-based network analysis

**DOI:** 10.1093/bioadv/vbag050

**Published:** 2026-02-15

**Authors:** Jaesub Park, Woochang Hwang, Seokjun Lee, Hyun Chang Lee, Méabh MacMahon, Matthias Zilbauer, Namshik Han

**Affiliations:** Cambridge Stem Cell Institute, University of Cambridge, Cambridge, CB2 0AW, United Kingdom; Milner Therapeutics Institute, University of Cambridge, Cambridge, CB2 0AW, United Kingdom; Cambridge Centre for AI in Medicine, Department of Applied Mathematics and Theoretical Physics, University of Cambridge, Cambridge, CB3 0WA, United Kingdom; CardiaTec Biosciences Ltd, Cambridge, CB2 1GE, United Kingdom; Cambridge Stem Cell Institute, University of Cambridge, Cambridge, CB2 0AW, United Kingdom; Milner Therapeutics Institute, University of Cambridge, Cambridge, CB2 0AW, United Kingdom; Cambridge Centre for AI in Medicine, Department of Applied Mathematics and Theoretical Physics, University of Cambridge, Cambridge, CB3 0WA, United Kingdom; Cambridge Stem Cell Institute, University of Cambridge, Cambridge, CB2 0AW, United Kingdom; Milner Therapeutics Institute, University of Cambridge, Cambridge, CB2 0AW, United Kingdom; Cambridge Centre for AI in Medicine, Department of Applied Mathematics and Theoretical Physics, University of Cambridge, Cambridge, CB3 0WA, United Kingdom; CardiaTec Biosciences Ltd, Cambridge, CB2 1GE, United Kingdom; Cambridge Stem Cell Institute, University of Cambridge, Cambridge, CB2 0AW, United Kingdom; Department of Paediatrics, University of Cambridge, Cambridge, CB2 0QQ , United Kingdom; Department of Paediatric Gastroenterology, Hepatology and Nutrition, Cambridge University Hospitals (CUH), Addenbrooke’s, Cambridge, CB2 0QQ, United Kingdom; Cambridge Stem Cell Institute, University of Cambridge, Cambridge, CB2 0AW, United Kingdom; Milner Therapeutics Institute, University of Cambridge, Cambridge, CB2 0AW, United Kingdom; Cambridge Centre for AI in Medicine, Department of Applied Mathematics and Theoretical Physics, University of Cambridge, Cambridge, CB3 0WA, United Kingdom; Department of Quantum Information, Institute for Convergence Research and Education in Advanced Technology and Engineering, Yonsei University, Seoul, 03722, Republic of Korea; Department of Nano Biomedical Engineering (NanoBME), Advanced Science Institute, Yonsei University, Seoul, 03722, Republic of Korea; Center for Nanomedicine, Institute for Basic Science (IBS), Seoul, 08826, Republic of Korea

## Abstract

**Motivation:**

Long COVID is a multisystem condition characterized by persistent symptoms such as fatigue, cognitive impairment, and systemic inflammation following COVID-19 infection. However, its mechanisms remain poorly understood. In this study, we applied the quantum walk, a computational approach leveraging quantum interference, to explore large-scale SARS-CoV-2–induced protein networks.

**Result:**

Compared to the conventional random walk with restart method, the quantum walk demonstrated superior capacity to traverse deeper regions of the network, uncovering proteins and pathways implicated in Long COVID. Key findings include mitochondrial dysfunction, thromboinflammatory responses, and neuronal inflammation as central mechanisms. Quantum walk uniquely identified the CDGSH iron-sulfur domain-containing protein family and VDAC1, a mitochondrial calcium transporter, as critical regulators of these processes. VDAC1 emerged as a potential biomarker and therapeutic target, supported by FDA-approved compounds such as cannabidiol. These findings highlight quantum walk as a powerful tool for elucidating complex biological systems and identifying novel therapeutic targets for conditions like Long COVID.

**Availability and implementation:**

The code and input data that were used for this study are available at https://github.com/Namshik-Han-Lab/QuantumWalk-LongCovid.

## 1 Introduction

Long COVID, also known as post-acute sequelae of SARS-CoV-2 infection, represents a complex constellation of persistent symptoms that continue or develop weeks to months after the initial COVID-19 infection (Davis *et al.* 2021, Nalbandian *et al.* 2021, Huang *et al.* 2023). The heterogeneous nature of Long COVID poses a substantial challenge to healthcare systems worldwide, as patients can experience vastly different combinations of symptoms with varying degrees of severity (Deer *et al.* 2021, Dennis *et al.* 2021, Soriano *et al.* 2022, Davis *et al.* 2023, Maurice 2024). Current computational approaches to Long COVID face challenges (Cau *et al.* 2022). Traditional methods inadequately capture complex symptom–pathway interactions (Wise 2021), while many models overlook temporal dynamics and patient-specific factors (Wang *et al.* 2024). This underscores the need for integrative computational frameworks that combine molecular, clinical, and patient-reported data to reveal hidden biological network patterns (Su *et al.* 2022).

In recent years, quantum algorithms, including QW, have shown great potential in efficiently navigating graph-based systems, offering insights that are challenging to obtain through classical methods. Quantum walk (QW) algorithms extend the concept of classical random walks by leveraging quantum mechanics principles such as superposition and interference (Venegas-Andraca 2012). This unique behavior makes QW especially suited for uncovering patterns in large, interconnected networks. Among the algorithms simulating QW, discrete-time quantum walk (DTQW) is particularly notable (Lovett *et al.* 2010). The stepwise unitary evolution of DTQW creates interference patterns, absent in classical random walk with restart (RWR), which may lead to fundamentally different outcomes in network analysis.

Building on the foundational principles of QW, recent research has explored its application in biological systems, demonstrating its ability to address challenges in network analysis and disease prioritization. For instance, QW has been utilized to rank network nodes, reveal community structures in complex networks, and perform real-world link prediction tasks (Chawla *et al.* 2020, Mukai and Hatano 2020, Liang *et al.* 2022, Liu *et al.* 2024). More recently, QW has also been applied to address a range of complex biological problems. For instance, predicting missing protein–protein interactions in incomplete PPI networks has been explored using hybrid approaches that combine continuous-time classical and quantum walks (Goldsmith *et al.* 2023). QW dynamics have also been proposed as a mechanistic basis for how proteins efficiently locate target DNA sequences, offering a quantum-inspired model for protein–DNA search strategies (D’Acunto 2021). Additionally, a QW-based disease gene prioritization framework operating on protein–protein interaction networks has demonstrated improved performance over traditional diffusion methods, including the approach introduced by Saarinen *et al.* (2024). Building on these efforts, Dubovitskii *et al.* (2025) further evaluated continuous-time quantum walks against random walk with restart across multiple biomolecular and cell–cell interaction networks, showing that quantum propagation can more sensitively capture biologically relevant structure and improve disease gene ranking (Dubovitskii *et al.* 2025). These examples highlight the potential of QW, positioning it as a promising tool in modern computational biology and suggesting that it could play a pivotal role in advancing the analysis of complex biological systems. However, although these approaches highlight the algorithmic advantages of QW, they largely focus on predictive performance and do not explicitly examine how QW dynamics translate into biological mechanisms or pathway-level interpretations.

In this study, we employed quantum-inspired approaches to analyze protein interaction networks associated with Long COVID, focusing on their ability to uncover critical biological mechanisms. Our results revealed that QW not only outperformed conventional methods in prioritizing validated Long COVID-associated proteins but also uncovered pathways closely tied to the disease’s pathophysiology, including mitochondrial dysfunction. Notably, QW extended its exploration into deeper regions of the network, uncovering critical biological interactions that underlie mitochondrial dysfunction—a hallmark of Long COVID. These findings highlight the potential of QW-based approaches in advancing our understanding of complex diseases such as Long COVID. By overcoming the limitations of traditional network analysis methods, QW offers a robust platform for uncovering novel disease mechanisms and facilitating the identification of therapeutic targets.

## 2 Methods

### 2.1 Disease-associated proteins

A total of 332 high-confidence SARS-CoV-2–human interactions were obtained from Gordon *et al.* (2020) (Table S3, available as supplementary data at *Bioinformatics Advances* online). A total of 332 high-confidence virus-host interactions were used as directly interacting proteins (DIPs). Differentially expressed SomaScan measurements in 6-month Long COVID patients versus recovered patients during acute COVID-19 were obtained from Cervia-Hasler *et al.* (2024). A total of 1335 proteins were extracted by combining Data S1 and S3, available as supplementary data at *Bioinformatics Advances* online and used as differentially expressed proteins (DEPs). The proteins that were significantly up- or down-regulated (two-tailed *t* tests, *P* < .05, |log2FC| > 0) were selected.

### 2.2 Long COVID network construction

**Figure 1:**
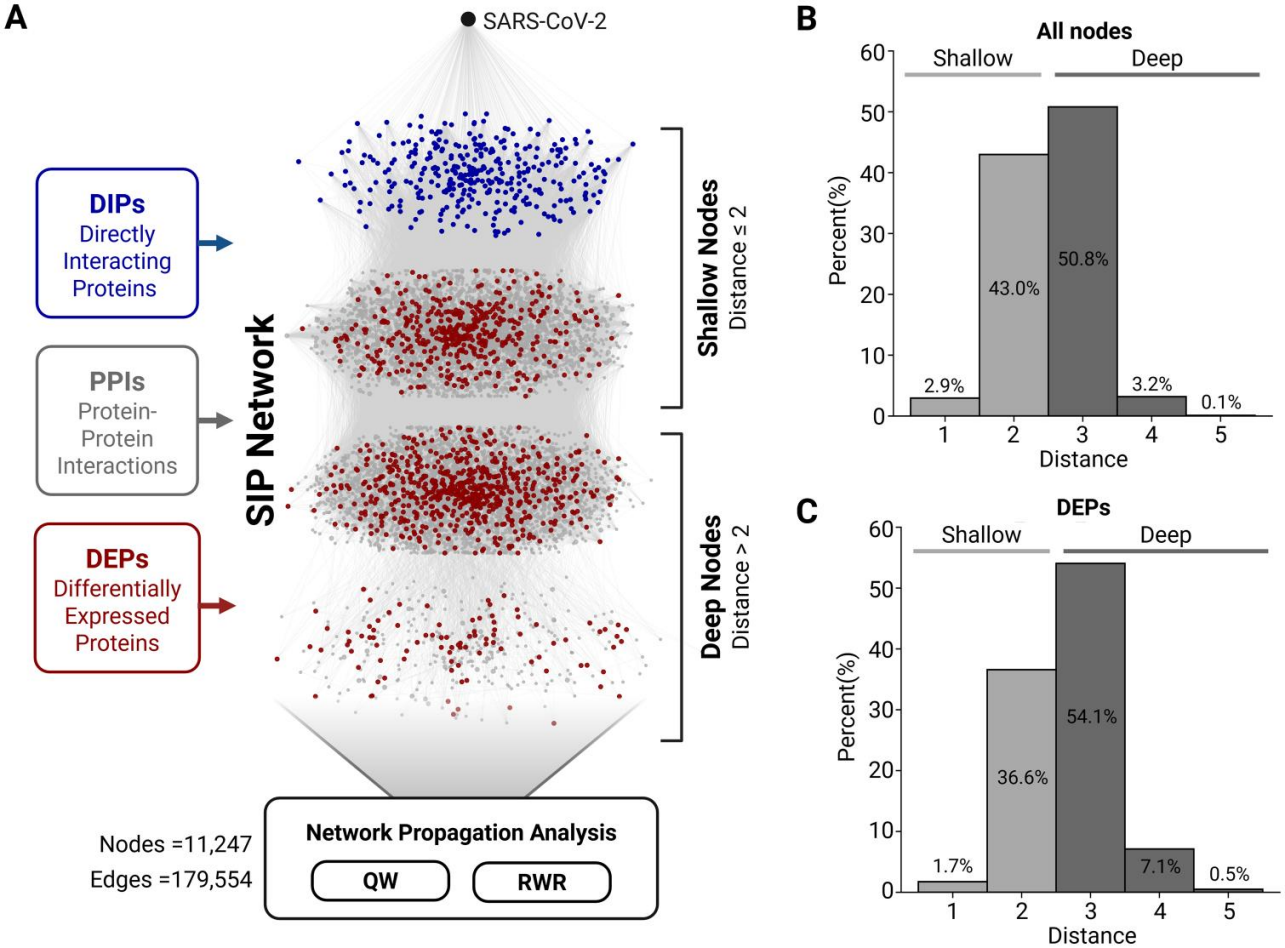
The SIP network distances show that deep exploration is needed to analyze paths to DEPs. (A) Schematic diagram of the SIP network construction process. The central network represents the constructed SIP network, with three clusters of nodes at the top corresponding to shallow nodes (distances 1, 2, and 3 from the SARS-CoV-2 node) and one cluster at the bottom representing deep nodes (distances ≥ 4). (B) Histogram showing the distance distribution of all nodes in the SIP network according to their distance from the SARS-CoV-2 node. (C) Histogram illustrating the distance distribution of DEPs in the SIP network from the SARS-CoV-2 node, highlighting their enrichment in deep regions.

The SIP network was constructed using a human protein–protein interaction (PPI) backbone sourced from the STRING database v11.5 (Szklarczyk *et al.* 2023). To ensure a balance between data reliability and network comprehensiveness, we filtered the interactions to include only those with a STRING-defined interaction score of ≥ 0.4 (medium confidence). This threshold was selected based on an empirical evaluation, which showed that it preserved connections for over 90% of all possible DIP–DEP pairs (*N* = 438 884), while higher thresholds (≥ 0.7 and ≥ 0.9) resulted in reduced coverage (Fig. 1A, available as supplementary data at *Bioinformatics Advances* online). This approach minimized selection bias and enabled the construction of an unbiased and comprehensive network. Subsequently, the final network topology was defined by identifying all shortest paths between the set of DIPs and the set of DEPs. These paths were computationally determined using the “all_shortest_paths” function from the NetworkX v3.4.2 (Hagberg *et al.* 2008), resulting in a layered network architecture comprising 11 247 nodes and 179 554 unique edges.

### 2.3 Long COVID proteins and prediction analysis

Differentially expressed SomaScan measurements at the 6-month follow-up in Long COVID patients versus recovered patients were obtained from Cervia-Hasler *et al.* (2024). A total of 514 proteins were extracted by combining Data S2 and S4, available as supplementary data at *Bioinformatics Advances* online, which were used as Long COVID proteins (LCPs). The proteins that were significantly up- or down-regulated (two-tailed *t* tests, *P* < .05, |log2FC| > 0) were selected. Among 514 LCPs, 423 LCPs were mapped onto the SIP network, and these are used for further analysis. The prediction performance for LCPs was primarily evaluated using the area under the precision-recall curve (AUPR), which is well-suited for imbalanced datasets with sparse positive labels (Davis and Goadrich 2006). The ground truth (positive set) consisted of the 423 LCPs mentioned earlier, while the total background (search space) was defined as the 4802 proteins measurable by SomaScan that are included in the SIP network. We also measured Precision at k (P@k) for the top 1%, 3%, 5%, and 10% of predictions to assess the methods’ effectiveness in retrieving top-ranked candidates, a critical factor for practical biomedical prioritization. To ensure a rigorous comparison, the RWR baseline was optimized via a grid search on the damping parameter alpha (ranging from 0 to 1 with a step size of 0.01), and the QW model was compared against the best-performing RWR configuration.

### 2.4 Network analysis

QW and RWR were employed to prioritize Long COVID-related proteins in the SIP network. The SARS-CoV-2 node was used as the starting node for both QW and RWR. RWR was implemented using the Personalized Pagerank algorithm from the Python package NetworkX v3.4.2 (Hagberg *et al.* 2008) (Information A, available as supplementary data at *Bioinformatics Advances* online). Parameters for RWR confirmed for sufficient convergence of a probability distribution (maximum iteration number: 100, EDP threshold: $1.0\times 10^{-5}$). The damping parameter alpha was set to 0.998, a value optimized via grid search to maximize the AUPR for LCP prediction (Figs 6A and 7F, available as supplementary data at *Bioinformatics Advances* online). For the quantum approach, we utilized the discrete time quantum walk model employing Grover coins and flip-flop shift operators (Ambainis *et al.* 2016, Cação *et al.* 2021, Cedzich *et al.* 2022). These algorithms were implemented using the Hiperwalk (v2.0b18) high-performance quantum walk simulator (Motta *et al.* 2023) (Information B, available as supplementary data at *Bioinformatics Advances* online). Permutation tests with 1000 iterations were conducted using random networks matching the SIP network’s degree distribution to determine significance for the RWR scores. Proteins with an empirical *P*-value <.01 were classified as key proteins for RWR (Weiskittel *et al.* 2022, Boyd *et al.* 2023). Distance analysis was performed using the Shortest Paths function of NetworkX. The single farthest node with a distance of six from the SARS-CoV-2 node was excluded from the histogram visualization.

### 2.5 Calculation of EDP and EDAP

To calculate the change in probability distributions at each iteration from the QW and RWR analysis results, the Euclidean distance of probability distribution was used.

Let G=(V, E) be the analyzed graph with |V|=n, and let v be a node of graph G. Then EDP at step t is defined as


$$EDP_{t}=\sqrt{\sum_{i=1}^{n}{\left(P_{t}(v_{i})-P_{t-1}(v_{i})\right)}^{2}}$$

Where $P_{t}(v_{i})$ is probability on the node $v_{i}$ at step t. To address the oscillatory behavior of QW, which does not converge, the average probability and EDAP of node v at step t is defined as


$$\begin{array}{c} \bar{P_{t}}(v)=\frac{1}{t+1}\sum_{k=0}^{t}P_{k}(v)\\ EDAP_{t}=\sqrt{\sum_{i=1}^{n}{\left({\bar{P}}_{t}(v_{i})-{\bar{P}}_{t-1}(v_{i})\right)}^{2}} \end{array}$$

where n is the total number of nodes in the network. Finally, the average mixing time is defined as


$$M_{\varepsilon }=\min\{T|\forall t\geq T\to \mathrm{EDA}P_{t}(V)\leq \varepsilon \}$$

where ε is an arbitrarily small value. We used a value of $1.0\times 10^{-5}$ in this analysis, based on our observation that the EDAP converged near this value. The calculated average mixing time for QW was 1212 iterations.

### 2.6 Over-representation analysis

Over-representation analysis (ORA) was performed using the R package clusterProfiler v4.14.4 (Xu *et al.* 2024). Two distinct ORAs were conducted using Gene Ontology (GO) and Reactome gene set databases. Reactome-based ORA of LCPs applied an adjusted *P*-value threshold of <.01 to identify significantly enriched pathways. Reactome-based ORA of 27 overlapping proteins between QW key proteins and deep LCPs applied an adjusted *P*-value threshold of <.01. Reactome-based ORA of QW and RWR key proteins extracted the top 10 enriched pathways based on adjusted *P*-values. GO-based ORA of the CISD subnetwork extracted the top 20 enriched pathways based on adjusted *P*-values.

### 2.7 Computation time measurement

To assess the practical computational efficiency, we measured the wall-clock time required for each algorithm to complete the specified number of iterations. All simulations were performed on a computing server equipped with an AMD EPYC 7702 64-Core Processor and 512 GB of RAM running Debian GNU/Linux 12. To ensure statistical reliability, the runtime measurements were repeated 10 times independently, and the results are reported as the mean ± standard deviation.

## 3 Results

### 3.1 The SARS-CoV-2–induced protein network reveals the importance of deep exploration

To elucidate the mechanisms underlying Long COVID, we investigated how SARS-CoV-2 impacts host proteins and pathways using a SARS-CoV-2–induced protein (SIP) network (Han *et al.* 2021, Hwang and Han 2022). The rationale for constructing the SIP network lies in the assumption, based on cause and effect, that proteins significantly affected during acute COVID-19 could contribute to the long-term effects observed in Long COVID.

The SIP network was constructed by integrating directly interacting proteins (DIPs), which directly interact with the SARS-CoV-2 protein, and differentially expressed proteins (DEPs), the latter identified from proteins exhibiting differential expression during acute COVID-19. To provide a comprehensive framework for exploring these integrations, the SIP network was designed with three distinct layers: the DIP layer, the DEP layer, and an intermediate hidden layer that bridges the two, consisting of 11 247 nodes and 179 554 unique edges. Topological analysis was consistent with a scale-free ($R^{2}=0.877$) and small-world topology, characterized by high clustering coefficients (C≈0.265), consistent with the high sparsity (density≈0.003), as is typical of biological interactomes (Fig. 4, available as supplementary data at *Bioinformatics Advances* online) (Stumpf *et al.* 2008). To ensure the construction of an unbiased and comprehensive network, edges were defined using medium-confidence (≥ 0.4) interactions (see Section 2 and Fig. 1A, available as supplementary data at *Bioinformatics Advances* online for details). All shortest paths between DIPs and DEPs were identified, ensuring that all potential interactions were captured within the network.

To assess the impact of SARS-CoV-2 on the SIP network, we applied network propagation methods, QW and RWR (Fig. 1A, details in Section 2). These methods allowed us to model how the viral signal influences proteins across the network. For further analysis, we categorized the network nodes into shallow (distance ≤ 2) and deep (distance > 2) regions, where distance refers to the distance from SARS-CoV-2, enabling us to explore the distinct roles of proximal and distal proteins. Our analysis revealed distinct patterns in the distribution of proteins across the SIP network, as shown in Fig. 1A. Among all nodes (Fig. 1B), the majority of proteins in the SIP network were classified as deep nodes, with 50.8% at distance 3, 3.2% at distance 4, and 0.1% at distance 5. Shallow nodes accounted for 45.9%, with 2.9% at distance 1 and 43.0% at distance 2. When focusing on DEPs (Fig. 1C), a higher proportion of DEPs were observed in deep regions compared to the general background distribution. Specifically, 61.7% of DEPs were located in deep nodes, with 54.1% at distance 3, 7.1% at distance 4, and 0.5% at distance 5, while only 38.3% are in shallow nodes, with 1.7% at distance 1 and 36.6% at distance 2.

This enrichment of DEPs, which we know are changing in response to COVID-19 infection, in deeper regions suggests that proteins further from the SARS-CoV-2 signal play an important role in mediating the broader effects of the virus. These findings highlight the need to investigate both shallow and deep regions of the SIP network to fully capture the direct and indirect impacts of SARS-CoV-2.

### 3.2 Contrasting algorithmic properties highlights fundamental differences between QW and RWR

Proteins that are highly connected and involved in multiple pathways within a protein interaction network may exert significant influence on the network and perform essential functional roles. To identify key proteins of COVID-19, we applied QW and RWR to the constructed SIP network. By designating the SARS-CoV-2 node as the starting point for both RWR and QW, we aimed to prioritize nodes frequently visited by walkers along the numerous paths spanning from DIPs to DEPs. Key proteins were defined based on statistical significance derived from the RWR analysis. Specifically, we selected significant proteins for RWR based on empirical *P*-values obtained from permutation tests and then applied the same number of top-ranked proteins for QW to ensure a consistent comparison between the two methods (details in Section 2). In total, 947 proteins met the significance threshold for RWR, and the same number (947) of top-ranked proteins was selected for QW. This approach is intended to uncover proteins central to COVID-19 that may significantly influence disease progression beyond the acute phase and contribute to the development of Long COVID.

To comprehensively assess the practical applicability and theoretical foundation of the QW approach, we conducted several analyses encompassing computational complexity, convergence characteristics, and measured wall-clock time. Our complexity analysis is grounded in the actual matrix operations performed by the libraries used in this study (Saad 2003). RWR exhibits high scalability due to its reliance on sparse matrix-vector multiplication in the current implementation. Its cost per iteration is O(|E|), where E is the number of edges. In contrast, the computational cost per iteration for QW’s classical simulation in the current implementation is higher, typically scaling as $O(|{E|}^{2})$ which suggests limited scalability on large-scale graphs. However, this limitation is caused by the overhead in the current implementation related to converting sparse matrices to dense matrices during core operations. Therefore, this overhead could potentially be reduced in future implementations by directly applying sparse matrix operations.

The total computation time is determined not just by the cost per iteration but also by the number of iterations. RWR is known to converge to the unique stationary distribution with the restart mechanism. However, QW exhibits dynamic fluctuation in a probability distribution throughout the iterative process, along with the lack of an inherent guarantee of convergence. Therefore, it is crucial to calculate the mixing time, which is the point at which the node probability distribution reaches a stable state, allowing the determination of the final probability distribution (Venegas-Andraca 2012). To address this, we quantified the change in node probabilities at each iteration by calculating the Euclidean distance of probability distribution (EDP) between consecutive iterations. For the RWR, EDP progressively decreased across successive steps, converging to a significantly small value (1.0 × 10^−5^) within just 10 iterations (Fig. 1B, available as supplementary data at *Bioinformatics Advances* online). Based on this observation, the mixing time was defined as the iteration at which the EDP reached 1.0 × 10^−5^. The probability distribution at the mixing time was used as the result of the RWR algorithm.

**Figure 2:**
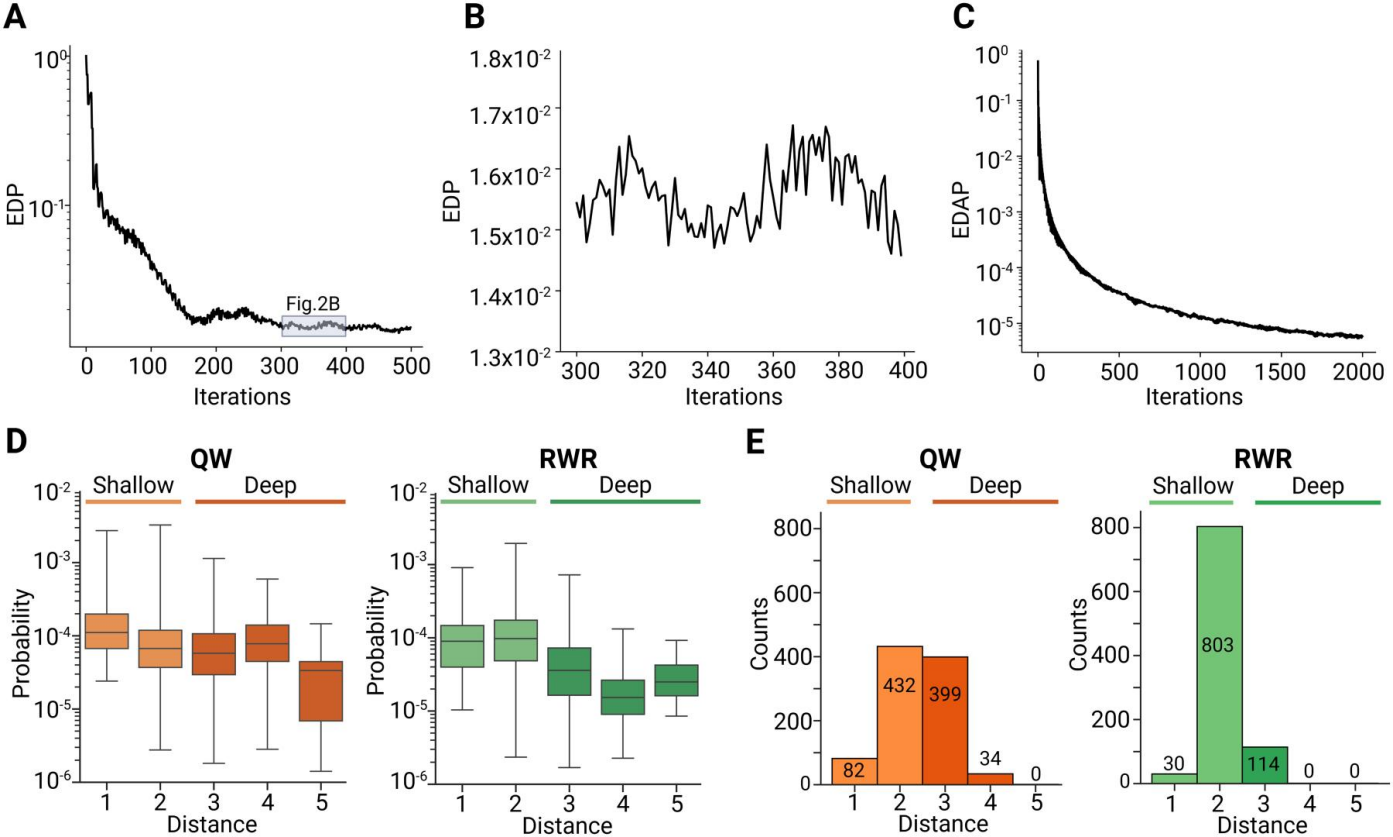
Analysis of QW and RWR on the SIP network reveals a fundamental difference in their exploration depth. (A) EDP at each iteration in QW, with the y-axis presented on a logarithmic scale to capture the wide dynamic range of changes. (B) EDP between the 300th and 400th iterations in QW, highlighting the oscillatory behavior that indicates non-convergence. (C) EDAP at each iteration in the QW, with the y-axis presented on a logarithmic scale. (D) Probability distribution of all nodes based on their distances from the SARS-CoV-2 node. The results of the QW are shown on the left, while those of the RWR are displayed on the right. (E) Histogram of top 947 nodes based on their distances from the SARS-CoV-2 node. These findings demonstrate QW’s ability to explore deeper regions of the network compared to RWR.0.

In contrast, the QW exhibited a decrease in the EDP initially but failed to converge to a sufficiently small value, instead oscillating around a constant value (Fig. 2A and B). To resolve this issue, we identified an approach that calculates an averaged probability distribution and defines the average mixing time (Lovett *et al.* 2010). As the iterations progressed in the QW, the Euclidean distance of averaged probability distributions (EDAP) between consecutive iterations gradually stabilized, eventually reaching below 1.0 × 10^−5^ (Fig. 2C). The average mixing time for QW was defined as the iteration where the change in EDAP fell below 1.0 × 10^−5^. The average probability distribution at the mixing time was used for subsequent analysis of QW results.

To further verify the robustness of our selected mixing time (t=1212), we performed an additional analysis using total variation distance relative to a long-term distribution ($t_{\max}=10\;000$) as an independent validation metric. This analysis confirmed that the selected mixing time represents an effective operational point, situated beyond the initial phase of rapid diffusion where the system begins to stabilize (Fig. 8A, available as supplementary data at *Bioinformatics Advances* online). Furthermore, we assessed the stability of the top 947 proteins by calculating the Jaccard Index and Spearman rank correlation relative to the $t_{\max}$ distribution. Both metrics exhibited rapid growth in the early stages and saturation as the system approached our selected time point (Fig. 8B and C, available as supplementary data at *Bioinformatics Advances* online). Notably, at the selected mixing time, the Jaccard Index reached 0.876 and the Spearman correlation reached 0.808, indicating high consistency with the long-term distribution despite the significant time difference (∼9000 steps). We also examined local variations within a window of t=1212 ± 100 and observed that both metrics remained consistently high (Jaccard Index > 0.975, Spearman correlation > 0.989) (Fig. 8D and E, available as supplementary data at *Bioinformatics Advances* online). These results confirm that the identified module is not an artifact of a specific time step but represents a stable structural property of the network.

Finally, we assessed the practical efficiency by measuring the wall-clock time spent by each algorithm when run for the specific number of iterations defined based on mixing time. In ten independent trials, the average computation time was 45.17 ± 0.38 s for the QW and 0.22 ± 0.05 s for the RWR on a computing server equipped with AMD EPYC 7702 and 512 GB of RAM (Fig. 5, available as supplementary data at *Bioinformatics Advances* online). Furthermore, when performing the same analysis on LCP subnetworks of varying sizes, we confirmed that the computation time increased as the number of edges grew, consistent with our mathematical complexity analysis. Although the computation time for the QW was higher than that of the RWR as expected, it remained within a practically feasible range.

### 3.3 QW enables comprehensive exploration of the SIP network beyond RWR’s limitations

After obtaining the results from each algorithm, we compared the characteristics of the walkers in QW and RWR by examining the distribution of probability values for each node in the SIP network based on their distance from the SARS-CoV-2 node (Fig. 2D). In the case of QW, the probability distribution for nodes at distances 1 to 4 from the SARS-CoV-2 node appeared nearly uniform, with only a slight decrease at distance 5. In contrast, RWR exhibited a clear decrease in probability values as the distance from the SARS-CoV-2 node increased. This observation is consistent with the well-documented limitation of RWR in effectively exploring nodes located farther from the starting point in large-scale networks. In contrast, the inherent properties of QW appear to overcome this limitation of RWR, enabling effective exploration of the deeper regions of the network.

**Figure 3:**
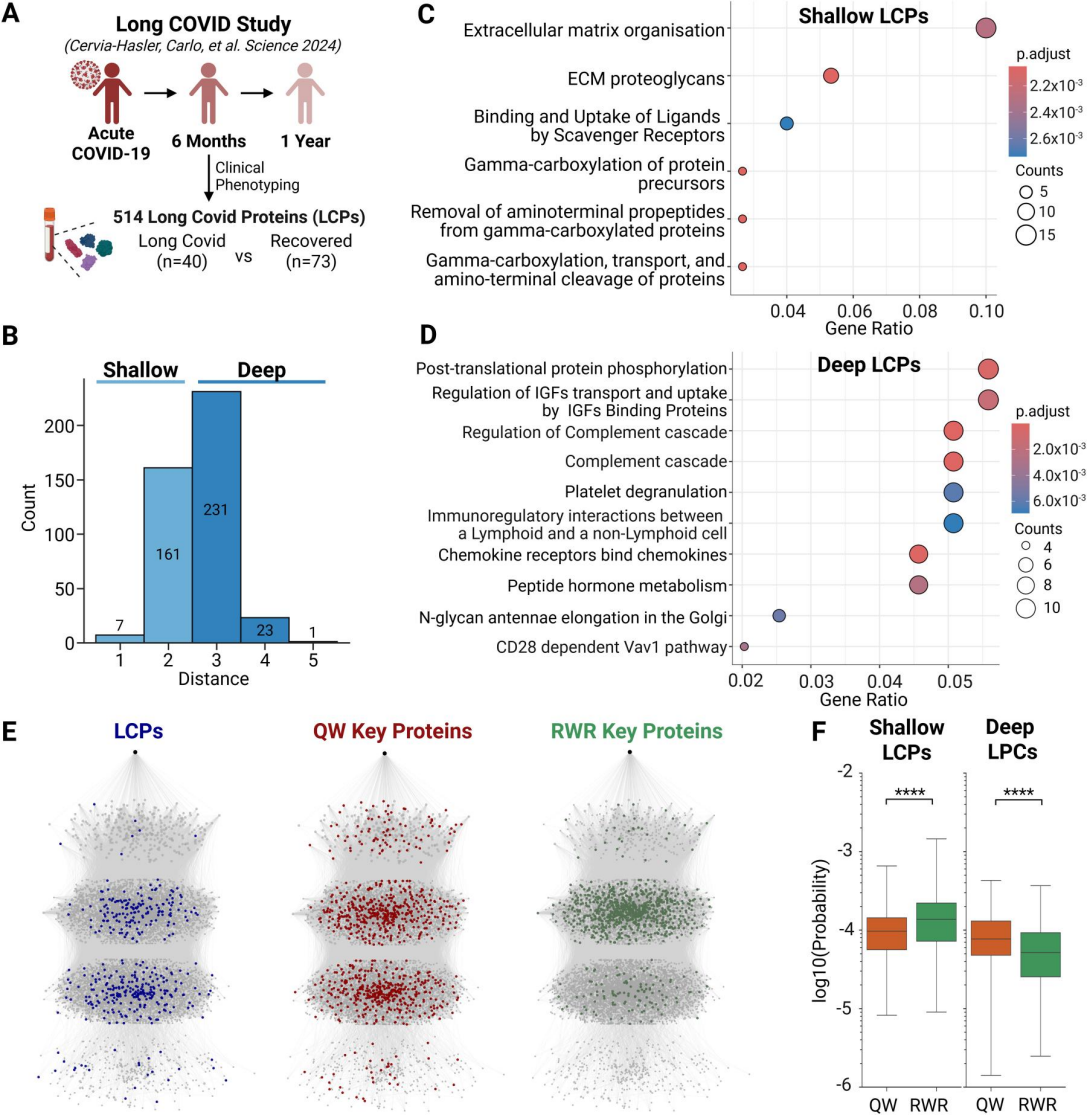
QW-identified key proteins closely reflect the spatial distribution of LCPs in the SIP network, outperforming RWR in identifying deep LCPs. (A) Schematic representation of a 12-month longitudinal proteomics study by Cervia-Hasler *et al*. (2024). Blood samples were collected at 6 months post infection from Long COVID patients (*n* = 40) and recovered COVID-19 patients (*n* = 73), leading to the identification of 514 differentially expressed proteins LCPs by comparing the two groups. (B) Spatial distribution of LCPs within the Long COVID network. Of the 423 mapped LCPs, 60% were positioned in deep nodes (distance >2) compared to 40% in shallow nodes (distance ≤2), reflecting their relevance to both acute and long-term disease processes. (C) Reactome-based ORA results for shallow LCPs, highlighting pathways such as ECM organization and gamma-carboxylation processes, linked to acute COVID-related processes. (D) Reactome-based ORA results for deep LCPs, highlighting pathways related to complement regulation and thromboinflammatory mechanisms, critical to Long COVID pathophysiology. (E) Visualization of the Long COVID network showing the spatial distribution of LCPs, QW key proteins, and RWR key proteins. Nodes are grouped into clusters based on their distance from the starting node (SARS-CoV-2): 1, 2, 3, and 4+. (F) Comparison of node probability for LCPs between QW and RWR across shallow and deep nodes. QW assigned significantly different probabilities to LCPs compared with RWR.

To further investigate the preceding results, we analyzed the distances from the SARS-CoV-2 node for the top 947 key proteins identified by each algorithm (Fig. 2E, details in Section 2). Most top nodes identified by QW were at distances of 2 or 3 from the SARS-CoV-2 node, with a smaller fraction at distance 4. Conversely, top nodes identified by RWR were primarily at distances of 1 or 2, with very few nodes at distance 3. These findings suggest that RWR outcomes are strongly distance-dependent, prioritizing proteins closer to the starting node. In contrast, QW assigns relatively uniform probabilities to nodes farther from the starting node, identifying them as key proteins as well. This fundamental difference between QW and RWR remained robust under variations in their respective key parameters (Figs 2A and B and 3A and B, available as supplementary data at *Bioinformatics Advances* online). Given that the SIP network represents a collection of paths from SARS-CoV-2 nodes to DEPs, and DEPs exist as far as distance 5, these results suggest that QW sufficiently explores the network more comprehensively and may offer a more accurate assessment of node importance within these pathways.

### 3.4 QW-identified key proteins show spatial similarity to LCPs in the SIP network

To evaluate the potential of QW in identifying proteins from the SIP network, which go on to be associated with Long COVID, high-confidence Long COVID proteins (LCPs) were curated from the literature. A recent study conducted a comprehensive 12-month longitudinal proteomics analysis to investigate changes in blood serum proteins in Long COVID patients (Fig. 3A) (Cervia-Hasler *et al.* 2024). Long COVID was defined as the persistence of one or more COVID-19–related symptoms (e.g. fatigue, pulmonary symptoms, gastrointestinal symptoms, cognitive symptoms, etc.) at six months post-infection, without alternative diagnosis. Among the 6596 human proteins analyzed, 514 differentially expressed proteins, referred to as LCPs, were identified at the 6-month follow-up by comparing Long COVID patients (*n* = 40) to recovered patients (*n* = 73). To determine the spatial distribution of LCPs within the SIP network, 423 of the 514 LCPs were mapped onto the network and categorized by their distances from the starting SARS-CoV-2 node (Fig. 3B). Most LCPs were positioned at distances 2 and 3 from the SARS-CoV-2 node, with 60% classified as deep LCPs (distance > 2) and the remaining 40% as shallow LCPs (distance ≤ 2). This distribution suggests that most LCPs reside in regions of the network further from DIPs of SARS-CoV-2.

To explore the functional roles of LCPs in shallow versus deep nodes, we performed over-representation analysis (ORA) on the two groups separately using Reactome database (Fig. 3C and D). For shallow LCPs, ORA revealed the enrichment in pathways such as gamma-carboxylation processes and extracellular matrix (ECM) organization. These pathways reflect acute COVID-related processes, as ECM degradation and inflammation-driven protease activity are characteristic of the disease (Narasaraju *et al.* 2024, Suski *et al.* 2024). In contrast, deep LCPs were enriched for pathways associated with complement regulation, thromboinflammatory mechanisms, and immune integrations, highlighting their direct relevance to Long COVID mechanisms (Gu *et al.* 2023, Cervia-Hasler *et al.* 2024). These findings demonstrate that shallow LCPs primarily reflect the general processes of COVID-19, while deep LCPs capture pathways that are mechanistically essential for the long-term effects of Long COVID.

In order to evaluate the ability of QW and RWR to identify LCPs across different network depths, we compared the spatial distributions of LCPs with those of the key proteins predicted by QW and RWR within the SIP network (Fig. 3E). Visualized networks revealed that the spatial distribution of QW key proteins closely resembled that of LCPs, whereas RWR key proteins exhibited a markedly different pattern. LCPs were predominantly located in deep nodes beyond the DIPs, a spatial distribution that was similarly reflected in QW key proteins. In contrast, RWR key proteins were concentrated near the DIPs in shallow nodes, with no representation at a distance of 4, further highlighting the limitations of RWR in exploring deep regions.

This disparity was further highlighted by the predicted probability distributions for all LCPs (Fig. 3F). While RWR assigned significantly higher probabilities to shallow LCPs compared to QW, QW assigned significantly higher probabilities to deep LCPs compared to RWR. This difference remained robust across changes in key algorithmic parameters, underscoring the consistency of the observed pattern (Figs 2C and 3C, available as supplementary data at *Bioinformatics Advances* online). Thus, QW demonstrated greater effectiveness in identifying deep LCPs, likely due to its enhanced capability to explore deeper regions of the network. Considering our finding that deep LCP-enriched pathways are directly implicated in the mechanisms of Long COVID, these results suggest that QW is more likely to identify key LCPs that are strongly associated with the pathophysiology of Long COVID.

### 3.5 QW outperforms RWR in uncovering long COVID mechanisms and enriched pathways

To quantitatively evaluate the predictive performance of QW and RWR in identifying LCPs, we employed the area under the precision-recall curve (AUPR) as the primary predictive metric. To ensure an unbiased comparison, we evaluated the performance of RWR across the full range of damping parameters and compared QW against the optimal RWR result. We found that QW achieved an AUPR of 0.1013, representing an improvement over the maximum AUPR obtained by RWR (0.0987) (Fig. 6A, available as supplementary data at *Bioinformatics Advances* online). Additionally, we calculated the Precision at k (P@k) for the top 1%, 3%, 5%, and 10% of predictions, given that identifying top-ranked candidates is critical for practical applications in the biomedical field. While QW outperformed RWR in the top 1% and 3%, RWR showed higher precision at the top 5%, and both methods exhibited the same precision at the top 10% (Fig. 6B–E, available as supplementary data at *Bioinformatics Advances* online). We also confirmed that both the absolute predictive performance and the performance gap between QW and RWR remain stable and are not sensitive to local fluctuations in hyperparameters (Fig. 7, available as supplementary data at *Bioinformatics Advances* online). Collectively, these results demonstrate that QW is fundamentally superior to RWR in predicting LCPs, particularly in prioritizing high-confidence candidates.

**Figure 4:**
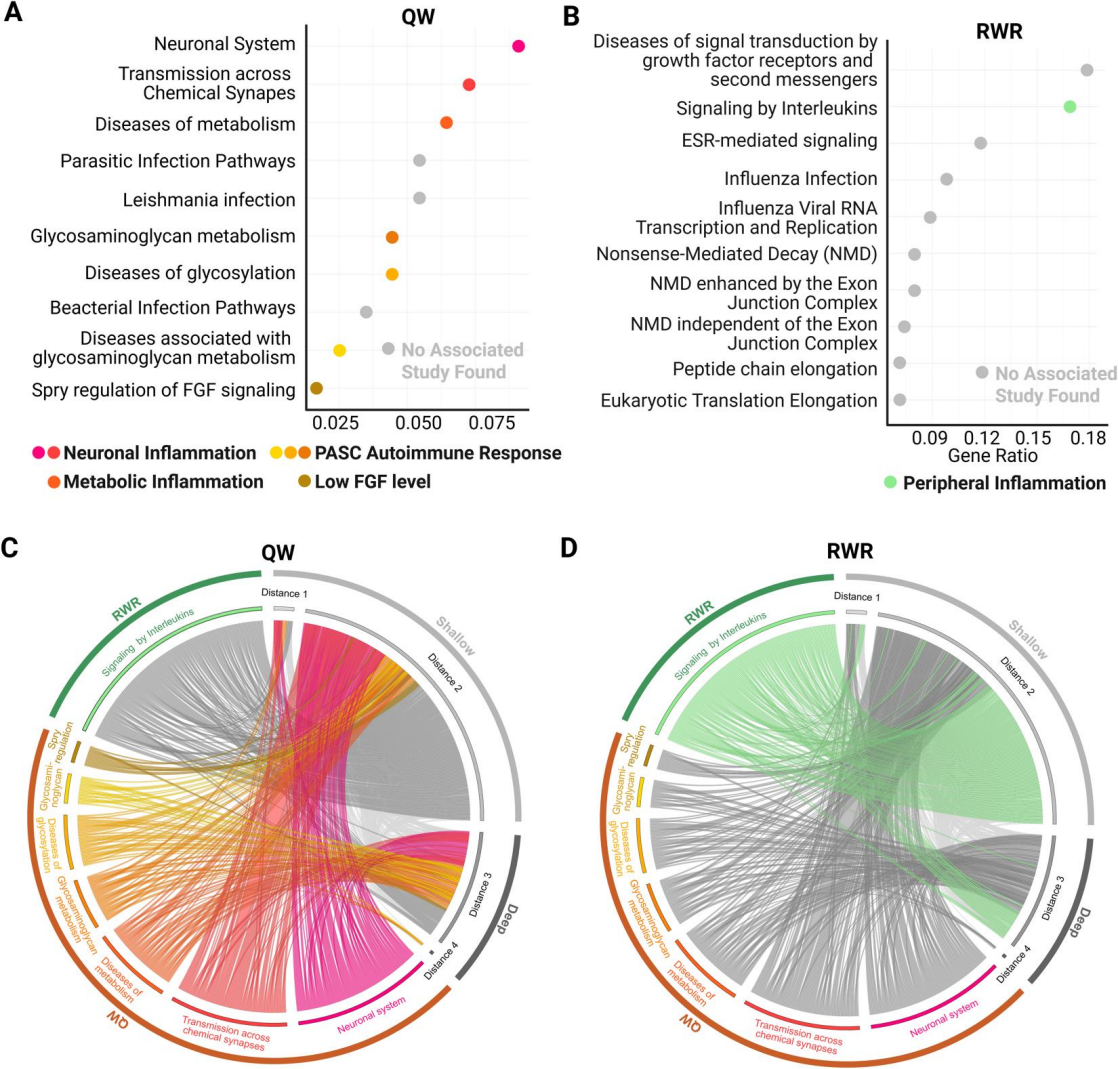
QW and RWR reveal distinct mechanisms underlying Long COVID symptoms, with QW demonstrating superior performance. (A) Reactome-based ORA results for the 947 key proteins of QW. Pathways are clustered based on their functional similarity, with many linked to Long COVID symptoms such as inflammation and metabolic dysregulation. (B) Reactome-based ORA results for the 947 key proteins of RWR (C and D) Each plot visualizes predicted key proteins and their enriched pathways, together with each protein’s network distance from the SARS-CoV-2 node. Proteins are arranged on the outer ring and grouped by topological distance. Radial links represent gene overlap—a connection is drawn when the same gene/protein belongs both to a given distance group and to the gene set of an enriched pathway. Pathways enriched by QW are shown in lower-left corner, whereas pathways enriched by RWR are shown in the upper-left corner, enabling comparison of method-specific enrichment patterns.

To assess the performance of QW and RWR in functional aspects, we compared the overlap between the 947 key proteins discovered by both methods and the LCPs located in shallow and deep nodes (Fig. 4A). In shallow nodes, QW identified 24 LCPs, whereas RWR identified 43, indicating that RWR achieved a higher number of predictions. The ORA of QW-predicted shallow LCPs revealed enrichment in Wnt/β-catenin signaling, apoptotic cleavage, and hormone metabolism pathways. In contrast, the pathways associated with RWR-predicted shallow LCPs included transcriptional regulation, MET activity, platelet activation, and cardiogenesis regulation (Fig. 1C and D, available as supplementary data at *Bioinformatics Advances* online). These results indicate that QW and RWR identify distinct biological functions within the shallow network. However, QW outperformed RWR in deep nodes by identifying 27 LCPs compared to only 5 predicted by RWR (Fig. 1E, available as supplementary data at *Bioinformatics Advances* online). ORA of these deep LCPs revealed enrichment in Long COVID-related pathways, including persistent dysregulation of the complement cascade, which contributes to thromboinflammation, chronic inflammation, and vascular injury (Gu *et al.* 2023, Baillie *et al.* 2024, Cervia-Hasler *et al.* 2024) (Fig. 1F, available as supplementary data at *Bioinformatics Advances* online). Additional enriched pathways included coagulation and fibrin clot formation, which are closely associated with hallmark Long COVID symptoms such as fatigue and post-exertional malaise (Gu *et al.* 2023, Cervia-Hasler *et al.* 2024). To further compare QW and RWR at the pathway level, we evaluated their ability to recover pathways strongly enriched in LCPs (Fig. 1G and H, available as supplementary data at *Bioinformatics Advances* online). Across both shallow and deep LCPs, QW consistently identified a greater number of LCP-enriched pathways than RWR, indicating superior sensitivity in capturing pathway-level signals. These results provide a clear comparison of how each method performs across shallow and deep regions of the network.

Subsequently, we performed enrichment analysis on the 947 key proteins identified by QW and RWR to assess their relevance to Long COVID (Fig. 4A and B). For each method, we prioritized the top 10 significantly enriched pathways and evaluated their biological relevance. Pathways captured by QW included those linked to neuronal inflammation (Spudich and Nath 2022, Hosp *et al.* 2024, Ryu *et al.* 2024), metabolic inflammation (Palmer *et al.* 2024), glycosylation disorders (Wang *et al.* 2022, Haslund-Gourley *et al.* 2023), glycosaminoglycan metabolism (Queisser *et al.* 2021), and FGF signaling dysregulation (Park *et al.* 2023). Collectively, these pathways demonstrate strong associations with autoimmune responses characteristic of Long COVID phenotypes (Long *et al.* 2023). These findings reinforce the relevance of QW in capturing key processes linked to immune dysregulation and persistent inflammation that underpin the pathophysiology of Long COVID (Mohandas *et al.* 2023). In contrast, RWR predominantly highlights pathways related to viral replication, nonsense-mediated mRNA decay (NMD), and immune responses (Angelini and Siciliano 2020). Notably, the interleukin signaling pathway plays multifaceted roles in modulating viral replication. Within this pathway, IL-6—a top-ranked node in the RWR analysis—promotes antiviral immune activation while simultaneously amplifying inflammatory responses (Wang *et al.* 2022). Crucially, this represents the sole pathway identified by RWR i.e. known to be associated with Long COVID, specifically through the process of peripheral inflammation (Ganesh *et al.* 2022, Yin *et al.* 2023).

Finally, to comprehensively characterize the topological relationship between key proteins within the Long COVID-related pathways identified by QW and RWR and their positions in the SIP network, we employed a circos plot. This visualization effectively highlights the distribution of key proteins enriched in pathways across shallow nodes, which are located closer to the SARS-CoV-2 node, and deep nodes, representing downstream and systemic effects of the viral perturbation. Consistent with our previous observations, QW-identified key proteins overrepresented in Long COVID-related pathways were broadly distributed across both shallow and deep nodes (Fig. 4C). In contrast, RWR-identified key proteins associated with Long COVID-related pathways were predominantly localized to shallow nodes, although they exhibited limited extension into deep nodes at distance 3 (Fig. 4D). Notably, deep nodes (distances 3 and 4) accounted for a significantly larger fraction of the QW-identified proteins (76%) compared to the RWR-identified proteins (28%). Consequently, this pathway-level analysis indicates that Long COVID-related pathways exhibit stronger associations with deep nodes.

Taken together, the RWR-enriched pathways highlight processes associated with the direct effects of viral infection and host responses, and this corresponds to their concentration in the shallower network regions, suggesting that RWR primarily captures immediate and localized interactions. In contrast, the QW-enriched pathways emphasize the systemic and long-range perturbations of COVID-19 on host biology, consistent with QW’s exploration of not only shallow but also deeper network regions. Furthermore, these findings align well with our previous observation that LCPs are evenly distributed across both shallow and deep network layers. Consequently, this underscores the critical importance of exploring deep nodes to uncover pathways associated with Long COVID and suggests that the superior capability of QW for deep exploration within the SIP network directly contributes to the derivation of biologically meaningful insights.

### 3.6 QW links mitochondrial dysfunction to long COVID mechanisms and identifies novel therapeutic targets

**Figure 5:**
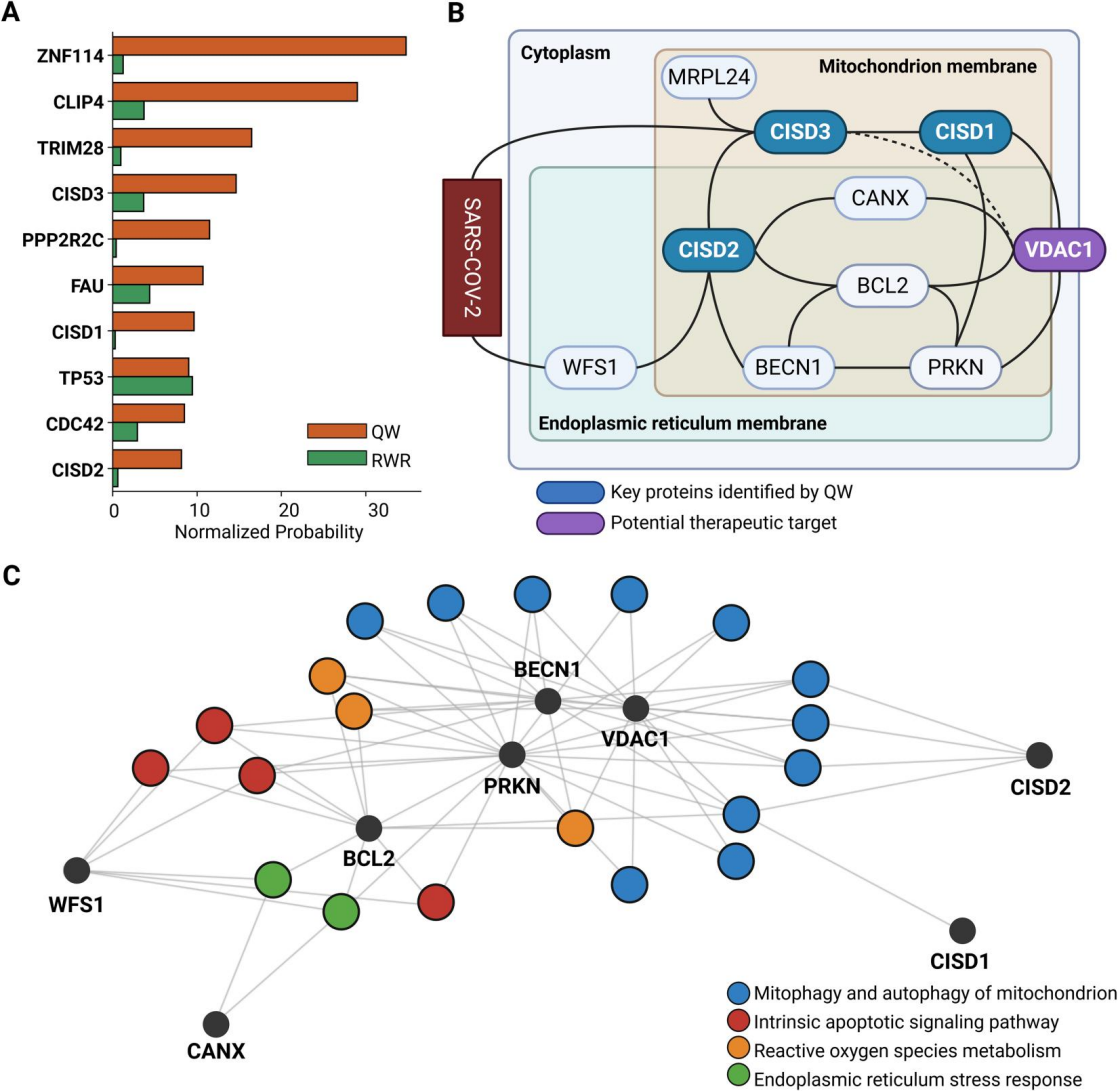
The top 10 QW-identified proteins reveal Long COVID mechanisms and therapeutic targets. (A) Z-normalized probability of the top 10 proteins predicted by QW in the SIP network. Results show that QW prioritizes most proteins significantly more than RWR. (B) Visualization of a sub-network comprising proteins directly connected to CISD1, CISD2, and CISD3 in the SIP network. The rectangular areas schematically represent the intracellular locations of proteins. Solid lines represent connections within the SIP network, while dashed lines denote additional direct associations confirmed through literature evidence. (C) The network visualization illustrates the linkages between proteins in the subnetwork and their associated biological processes, based on the top 20 results from Gene Ontology (GO)-based ORA of these proteins. Biological processes associated with the proteins are clustered to represent functional similarity. Processes include autophagy, mitophagy, ROS metabolism, and ER stress response, emphasizing their relevance to Long COVID mechanisms.

To assess the ability of the QW approach to propose specific Long COVID mechanisms from the SIP network and further identify novel therapeutic biomarkers, we examined the top 10 proteins predicted by QW (Fig. 5A and Table 2, available as supplementary data at *Bioinformatics Advances* online). Interestingly, the probability values for all proteins, except for TP53, were significantly lower in RWR compared to QW, emphasizing that these prioritized proteins were uniquely identified through the QW approach. Of particular interest was the inclusion of CISD1, CISD2, and CISD3 proteins from the CDGSH iron-sulfur binding domain-containing family in the list (Sengupta *et al.* 2018). Figure 5A highlights the significant difference in normalized probabilities between QW and RWR, with QW strongly prioritizing CISD family proteins. Notably, CISD1, CISD2, and CISD3 are particularly involved in regulating reactive oxygen species (ROS) metabolism (Lipper *et al.* 2018). The specific association between ROS metabolism and iron-sulfur clusters with COVID-19 and Long COVID is of significant interest, as multiple reports have highlighted these connections (Maio *et al.* 2021, Noonong *et al.* 2023, Xie *et al.* 2024).

We focused on this protein family and extracted a subnetwork of proteins directly connected to the CISD1, CISD2, and CISD3 proteins in the SIP network (Fig. 5B). The subnetwork was highly interconnected, with most of the proteins localized in the mitochondrion membrane and endoplasmic reticulum (ER) membrane, suggesting that the interactions and functions of the proteins are closely related to mitochondria-associated membranes (MAMs). Figure 5B provides a schematic view of the intracellular locations of these proteins, highlighting the dense connectivity within the MAMs. WFS1, CISD2, BCL2, VDAC1, and CANX are involved in Ca^2+^ regulation within MAMs (Gutiérrez *et al.* 2020, Loncke *et al.* 2021). Dysregulated mitochondrial Ca^2+^ can cause mitochondrial collapse or trigger mitophagy (Rimessi *et al.* 2013, Perrone *et al.* 2023). BECN1 and PRKN have a role in regulating mitophagy, with BECN1 promoting autophagosome formation at MAMs and PRKN facilitating mitochondrial ubiquitylation and recruitment of ubiquitin-binding receptors (Choubey *et al.* 2014, Heo *et al.* 2019, Quiles *et al.* 2023).

To further explore the overall biological processes associated with these proteins, we performed GO-based ORA, revealing significant enrichment in autophagy, mitophagy, ROS metabolism, and endoplasmic reticulum stress response (Fig. 5C and Table 3, available as supplementary data at *Bioinformatics Advances* online). Thus, an intensive literature review of each individual protein, coupled with computational enrichment analyses of all identified proteins, strongly supports the association between the QW subnetwork and mitochondrial dysfunction and mitophagy. Indeed, mitochondrial dysfunction and mitophagy have been identified as key factor in the pathogenesis of Long COVID (Chen *et al.* 2023, Madsen *et al.* 2024, Molnar *et al.* 2024).

Finally, we investigated whether QW could specifically identify drug targets for Long COVID. Within the subnetwork shown in Fig. 5B, VDAC1 emerged as a potential target due to its critical role in mitochondrial Ca^2+^ transport and its significant reduction in Long COVID patients (Peluso *et al.* 2022, Espín *et al.* 2023). The key function of the subnetwork we identified was the regulation of Ca^2+^ at the MAMs, with particular emphasis on VDAC1, a voltage-dependent anion channel protein that directly participates in mitochondrial Ca^2+^ transport. To evaluate VDAC1 as a potential therapeutic target, we investigated compounds targeting VDAC1 using the DrugBank database. Among the four identified compounds, cannabidiol is the only FDA-approved drug. Notably, the potential of cannabidiol as a therapeutic agent for Long COVID has been explored, leveraging its neuropsychiatric properties and anti-inflammatory effects (Esposito *et al.* 2020, Scott *et al.* 2023, Thurgur *et al.* 2024).

## 4 Discussion

Although from a public health perspective, COVID-19 has transitioned from a pandemic to an endemic disease, many patients still suffer from Long COVID, and clear solutions remain unavailable. In this study, we demonstrate that QW offers a deeper exploration of the global structure of large-scale SIP networks compared to RWR, enabling more precise identification of proteins and pathways implicated in Long COVID pathophysiology. Additionally, QW-based analyses highlight its potential for uncovering crucial disease pathways (mitochondrial dysfunction) and novel therapeutic targets, advancing our understanding of the Long COVID mechanisms. These findings underscore the potential of QW as a robust computational approach for investigating complex biological systems, positioning it as a promising tool for the study of multisystem disorders such as Long COVID.

A key limitation of conventional network methods such as RWR is their bias toward proximal nodes, limiting their ability to explore deeper network regions. In contrast, QW leverages quantum interference to more effectively traverse distal and systemic areas of the network. This capability is particularly relevant for conditions like Long COVID, where key biological processes are likely dispersed across broader network layers. Consistent with this, our analysis shows that QW more accurately prioritized LCPs, with predicted nodes exhibiting significantly higher concordance with LCP distributions than those identified by RWR. Comparative pathway enrichment revealed that QW, unlike RWR, prioritized pathways associated with thromboinflammation, neuroinflammation, and mitochondrial dysfunction—processes implicated in Long COVID symptoms such as fatigue, cognitive impairment, and systemic inflammation. Notably, mitochondrial dysfunction also emerged as a link between the QW-derived subnetwork and Parkinson’s disease, with PRKN mutations being a known cause (Hattori and Mizuno 2004). This observation aligns with recent evidence suggesting that COVID-19 may increase the risk of Parkinson’s disease via shared mechanisms such as mitochondrial impairment and immune dysregulation (Sulzer *et al.* 2020, Polverino *et al.* 2024).

Our analysis identifies VDAC1 as a potential therapeutic target for Long COVID, based on its critical role in mitochondrial Ca^2+^ regulation. Previous studies reported altered VDAC1 expression in Long COVID patients, supporting its biomarker potential (Espín *et al.* 2023). We expand on these findings by linking VDAC1 to mitochondrial dysfunction and systemic inflammation, emphasizing its therapeutic relevance. VDAC1 has also been extensively studied in inflammation-related and neurodegenerative diseases such as Alzheimer’s disease (Camara *et al.* 2017, Hu *et al.* 2022, Yang *et al.* 2024). Considering symptomatic overlaps with Long COVID, targeting VDAC1 may offer clinical benefits. Nevertheless, further experimental validation—such as in vitro or in vivo studies assessing VDAC1’s impact on mitochondrial Ca^2+^ flux or apoptosis, and patient-derived cellular or longitudinal multi-omics analyses—is essential to elucidate its mechanistic role. Given the scarcity of clinically approved VDAC1 modulators, drug repurposing strategies may provide a viable route to develop biomarker-guided therapies for Long COVID symptom management.

While this study demonstrates the utility of QW in Long COVID research, several limitations warrant consideration. The constructed protein interaction network relies on available databases and may not fully capture the dynamic and context-specific interactions underlying Long COVID. Incorporating longitudinal and multi-omics data could further enhance the accuracy and relevance of the network. In addition, the quantum walk framework used in this study relies on idealized simulations. Current quantum devices still face constraints in circuit depth and error rates, meaning that the behavior of QW on real hardware may diverge from simulated outcomes. A more detailed examination of hardware-related feasibility, as well as strategies to mitigate noise, will be important for translating these methods toward practical quantum implementations. Future work should also explore how the choice of operators and parameters shapes QW behavior across different biological networks and integrate QW with experimental approaches, such as proteomics and functional assays, to validate and extend these findings.

This study underscores the transformative potential of QW in advancing our understanding of Long COVID. By enabling deeper exploration of biological networks, QW has identified novel proteins and pathways that are critically involved in Long COVID pathophysiology. In particular, its ability to prioritize mitochondrial dysfunction and systemic processes further establishes its value in studying multisystem disorders or other post-acute sequelae, such as those following influenza, Ebola, or dengue (Bannister 1988, Choutka *et al.* 2022, Gandhi 2024). These findings not only enhance our understanding of this complex condition but also pave the way for the development of targeted therapies. As computational biology continues to evolve, approaches like QW hold promise for addressing the challenges posed by multifaceted diseases, ultimately contributing to more effective and personalized healthcare solutions.

## Supplementary Material

vbag050_Supplementary_Data

## Data Availability

All data needed to evaluate the conclusions in the paper are present in the paper and/or the Supplementary Materials. The code and input data that were used for this study are available at https://github.com/Namshik-Han-Lab/QuantumWalk-LongCovid. Additional data related to this paper may be requested from the authors.
