# Supplementary material for: Advancing understanding of long COVID pathophysiology through quantum walk-based network analysis
Source: Bioinform Adv. 2026 Feb 15;6(1):vbag050. doi: 10.1093/bioadv/vbag050 (PMC12975004; doi:10.1093/bioadv/vbag050)
Supplement: vbag050_Supplementary_Data [file vbag050_supplementary_data.docx]

**Advancing Understanding of Long COVID Pathophysiology**  **Through Quantum Walk-Based Network Analysis**

**[Supplementary Data]**

**Jaesub Park^1,2,3,†^, Woochang Hwang^4,†^, Seokjun Lee^1,2,3,‡^, Hyun Chang Lee^1,2,3,‡^, Méabh MacMahon^4^, Matthias Zilbauer^1,5,6^ and Namshik Han^1,2,3,7,8,9,*^**

**1 Cambridge Stem Cell Institute, University of Cambridge, Cambridge, UK**

**2 Milner Therapeutics Institute, University of Cambridge, Cambridge, UK**

**3 Cambridge Centre for AI in Medicine, Department of Applied Mathematics and Theoretical Physics, University of Cambridge, Cambridge, UK**

**4 Cardiatec Biosciences Ltd, Cambridge, UK**

**5 Department of Paediatrics, University of Cambridge, Cambridge, UK**

**6 Department of Paediatric Gastroenterology, Hepatology and Nutrition, Cambridge University Hospitals (CUH), Addenbrooke's, Cambridge, UK**

**7 Department of Quantum Information, Institute for Convergence Research and Education in Advanced Technology and Engineering, Yonsei University, Seoul, Republic of Korea**

**8 Department of Nano Biomedical Engineering (NanoBME), Advanced Science Institute, Yonsei University, Seoul, Republic of Korea**

**9 Center for Nanomedicine, Institute for Basic Science (IBS), Seoul, Republic of Korea**

**†These authors contributed equally.**

**‡These authors contributed equally.**

***Corresponding author. Email: nh417@cam.ac.uk.**

**
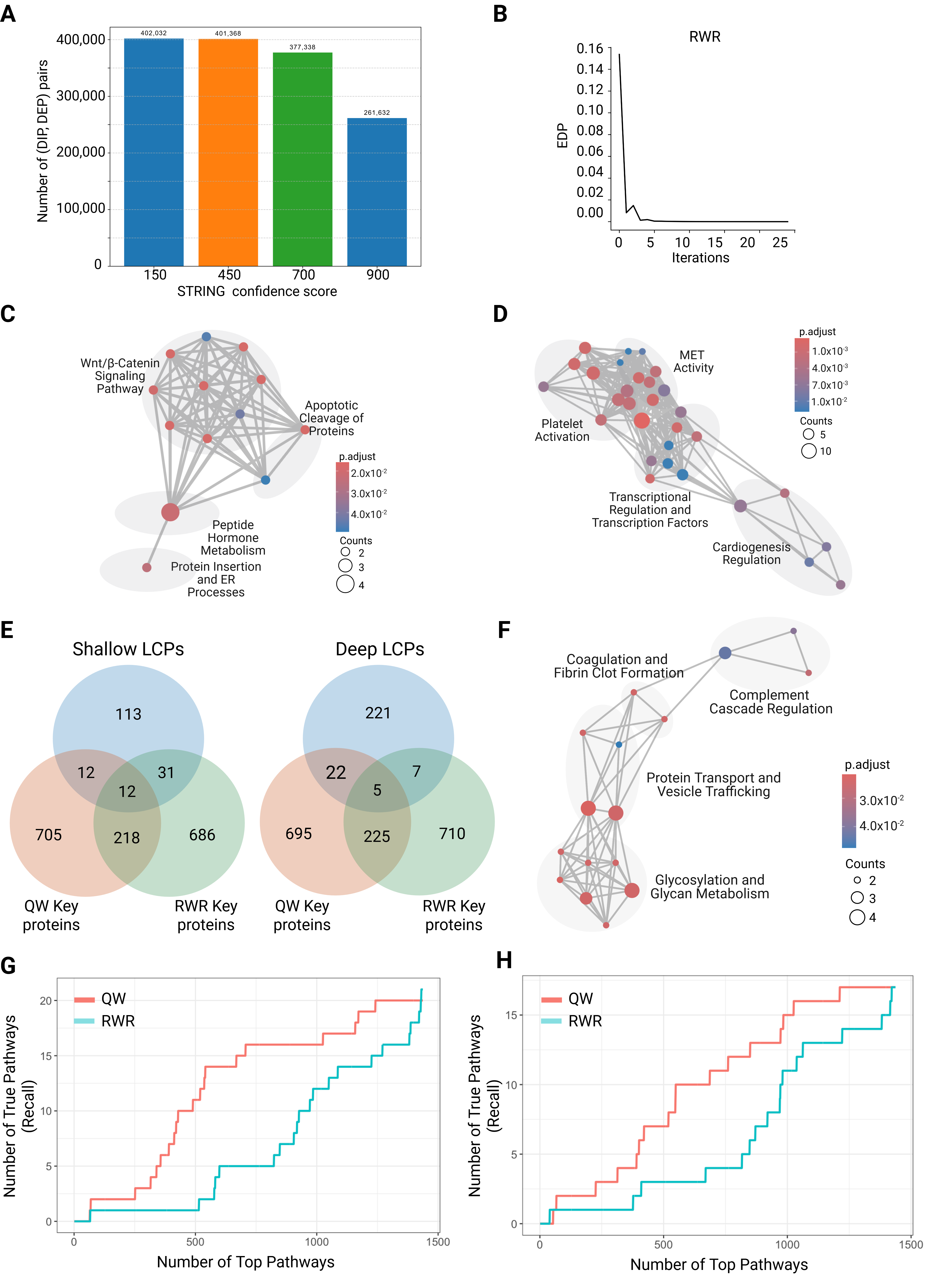
**

**Supplementary Figure 1. (A)** The y-axis indicates the number of DIP–DEP protein pairs connected at each STRING-defined interaction score threshold (x-axis): low (≥ 0.15), medium (≥ 0.4), high (≥ 0.7), and highest (≥ 0.9). The medium-confidence threshold (≥ 0.4) enabled the construction of an unbiased and comprehensive SIP network, with broad network coverage and minimal selection bias. **(B)** EDP at each iteration in RWR (alpha 0.85). RWR reaches a sufficiently low EDP (${1.0 \times10}^{-5}$) within just 10 steps **(C)** Reactome-based ORA results for the shallow LCPs identified by QW. **(D)** Reactome-based ORA results for the shallow LCPs identified by RWR. **(E)** Venn diagram of overlapping proteins between shallow and deep LCPs, QW key proteins, and RWR key proteins. QW uniquely identified 27 deep nodes overlapping with LCPs, which were not detected by RWR. **(F)** Reactome-based ORA results for the 27 deep LCPs uniquely identified by QW. Pathways sharing 20% of proteins are connected by edges. **(G, H)** Overlap analysis between LCP ORA and QW- or RWR-derived ORA results. The graphs display the number of pathways overlapping with the Reactome-based LCP ORA results (y-axis) as a function of the number of top-ranked pathways selected from QW or RWR analysis (x-axis). Results are shown for the shallow (G) and deep (H) datasets.


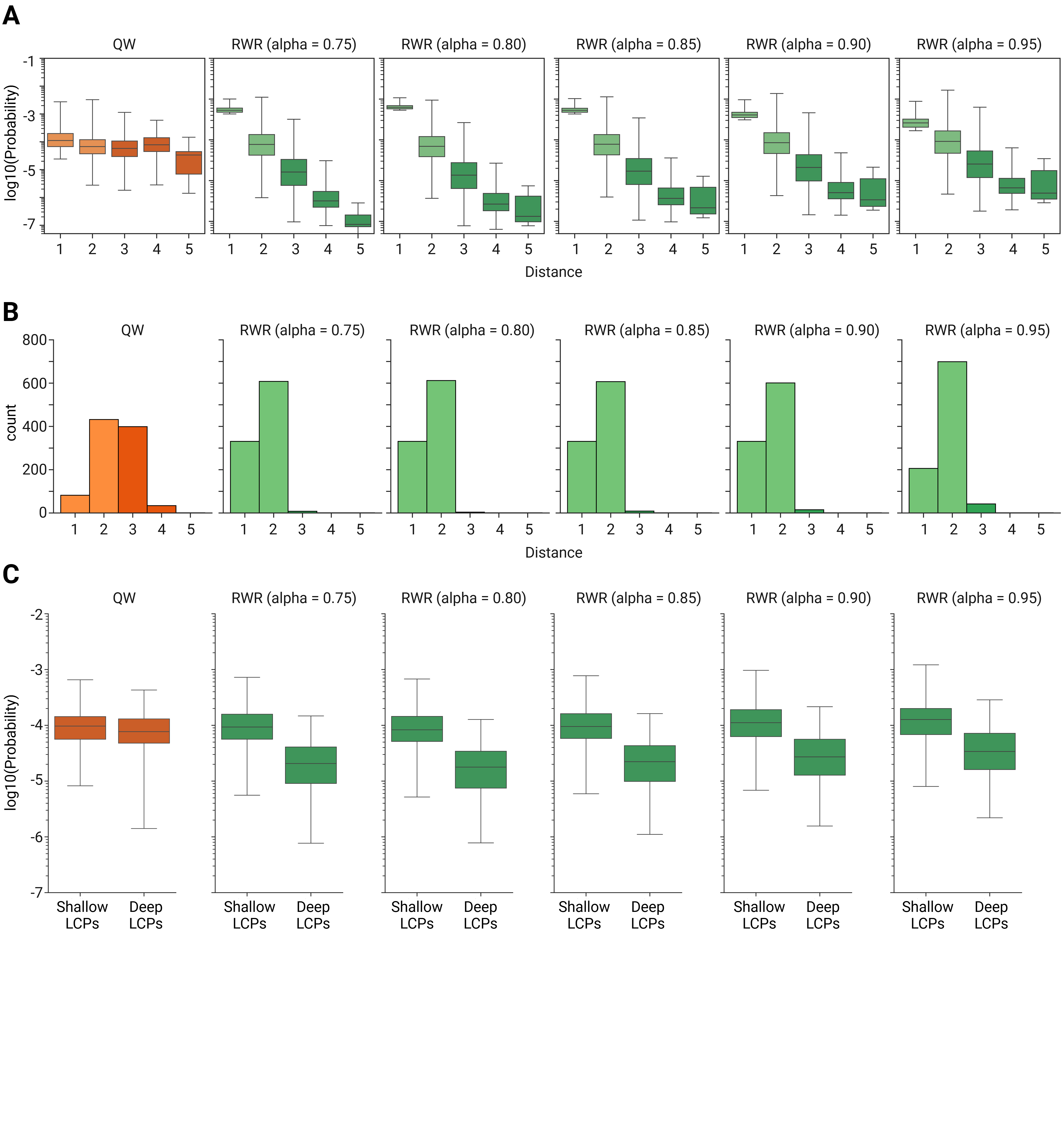


**Supplementary Figure 2.** **Robustness of RWR results with respect to variations in the alpha value.** The QW results are visualized in orange, while the RWR results are shown in green. The five distinct RWR plots represent results obtained by varying the alpha value from 0.75 to 0.95 in increments of 0.05. Changes in the alpha value did not significantly affect the overall pattern of the RWR results, and the fundamental differences between RWR and QW remained consistent. **(A)** Probability distribution of all nodes based on their distances from the SARS-CoV-2 node. **(B)** Histogram of top 947 nodes based on their distances from the SARS-CoV-2 node. **(C)** Comparison of node probability for LCPs between QW and RWR across shallow and deep nodes.

**
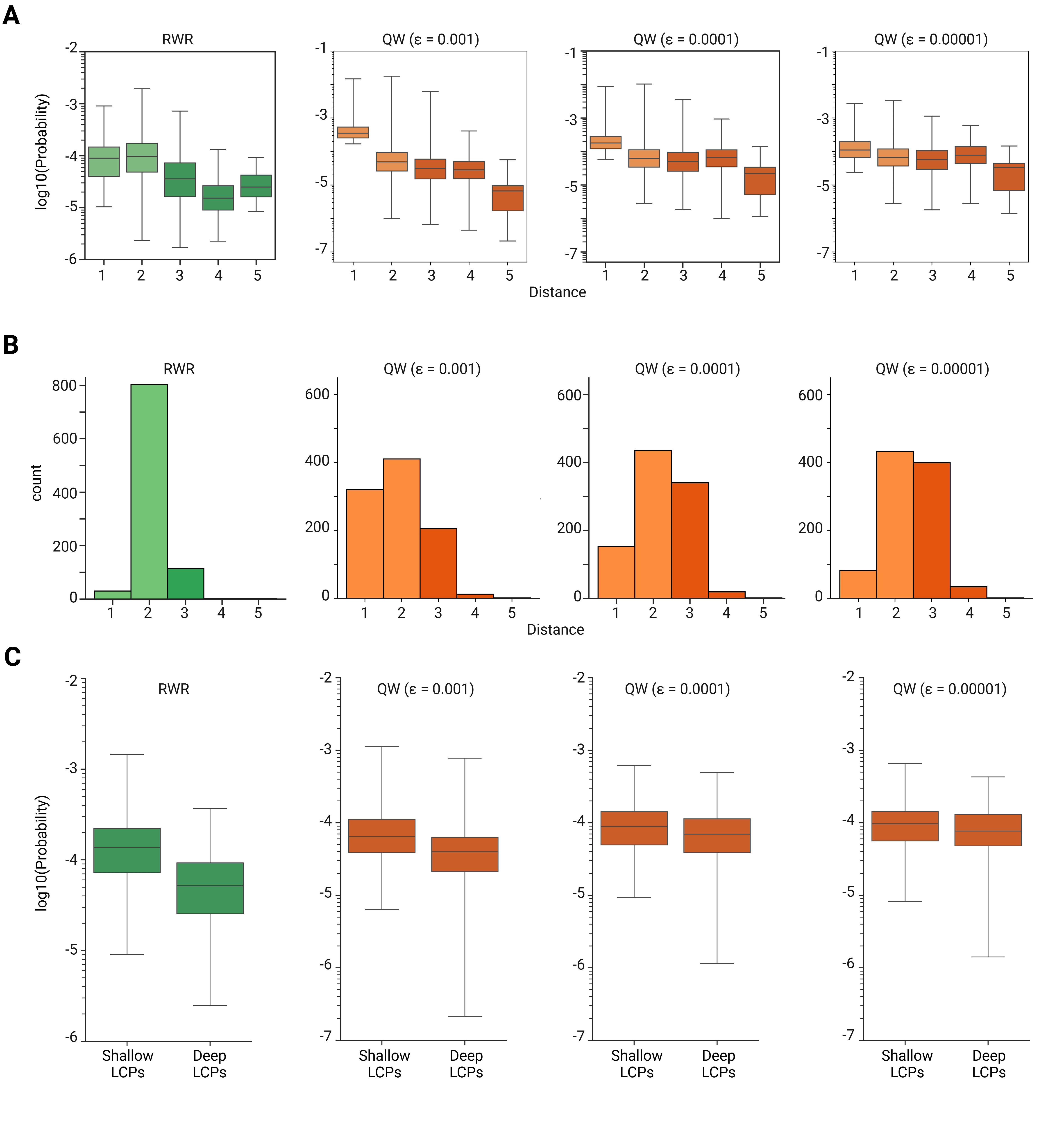
**

**Supplementary Figure 3. Robustness of QW results with respect to variations in the threshold for mixing time calculation.** The QW results are visualized in orange, while the RWR results are shown in green. The 3 distinct QW plots represent results obtained by varying the threshold $\varepsilon$ for mixing time calculation from 10x10^-3^ to 10x10^-5^ in increments 10 times. **(A)** Probability distribution of all nodes based on their distances from the SARS-CoV-2 node. **(B)** Histogram of top 947 nodes based on their distances from the SARS-CoV-2 node. **(C)** Comparison of node probability for LCPs between QW and RWR across shallow and deep nodes.

Changes in the threshold on an exponential scale did not significantly affect the overall pattern of the QW results, and the fundamental differences from RWR remained consistent. Notably, QW outperformed RWR in predicting deep LCPs even at early, non-converged stages, with this advantage becoming more pronounced as the algorithm approached its true convergence point. This highlights not only the robustness of the QW algorithm but also the validity and suitability of our approach to estimating and applying the mixing time.


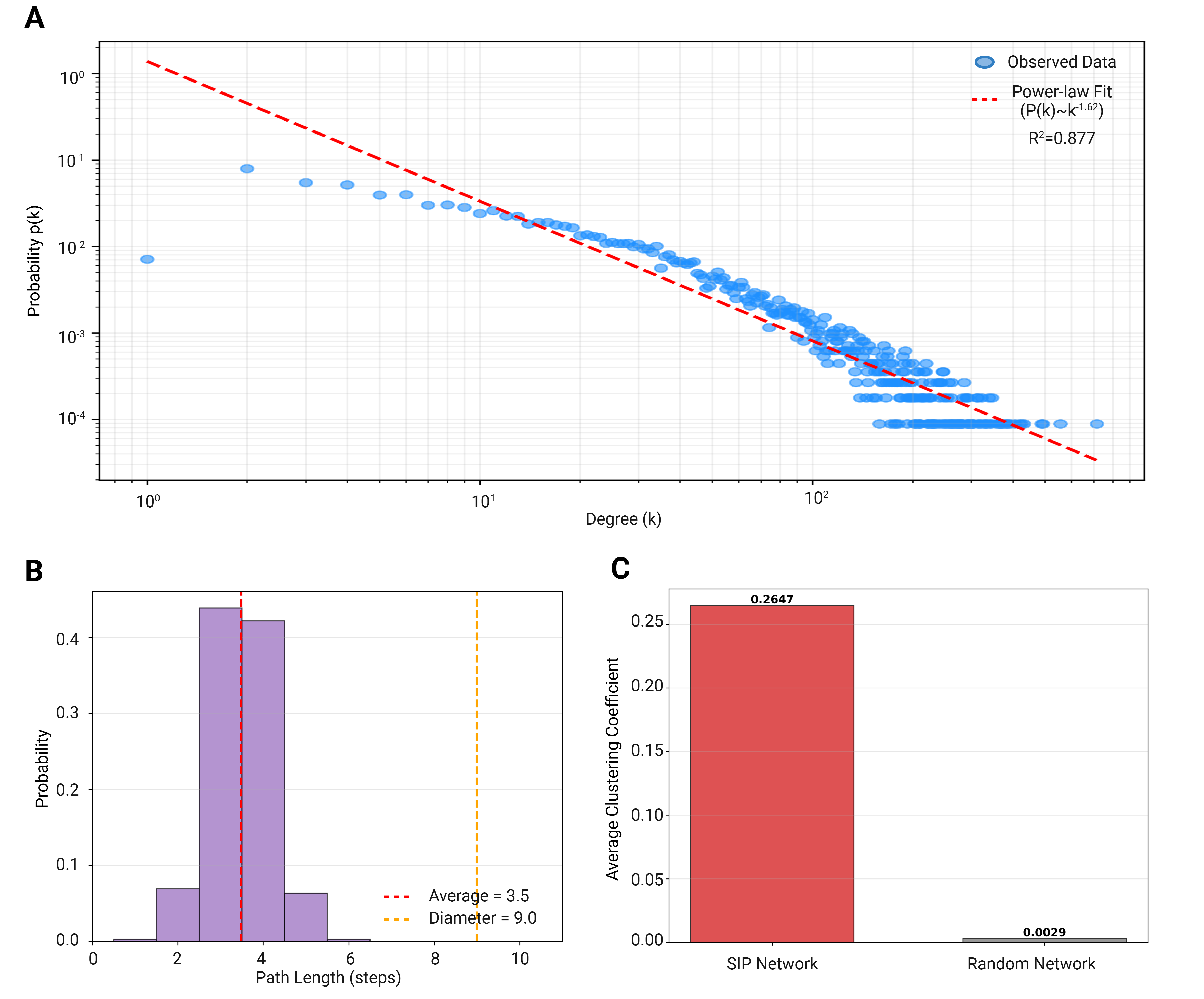


**Supplementary Figure 4.** **Topological characteristics of the Long COVID SIP network.** The structural properties of the constructed SIP network were analyzed to validate its biological relevance. (A) Degree probability distribution P(k) shown on a log-log scale. The observed data (blue dots) closely follows a power-law fit (red dashed line; R^2^ = 0.877), confirming a scale-free topology driven by highly connected hub nodes. (B) Distribution of shortest path lengths between all node pairs. The network exhibits efficient global connectivity, characterized by a short average path length (~3.50; red dashed line) and a small network diameter (9; orange dashed line). (C) Small-worldness test comparing the average clustering coefficient of the SIP network to that of random networks generated with the same size and density. The SIP network displays a significantly higher clustering coefficient (0.2647) compared to the random baseline (0.0029), confirming robust small-world properties and high local cohesiveness.

**
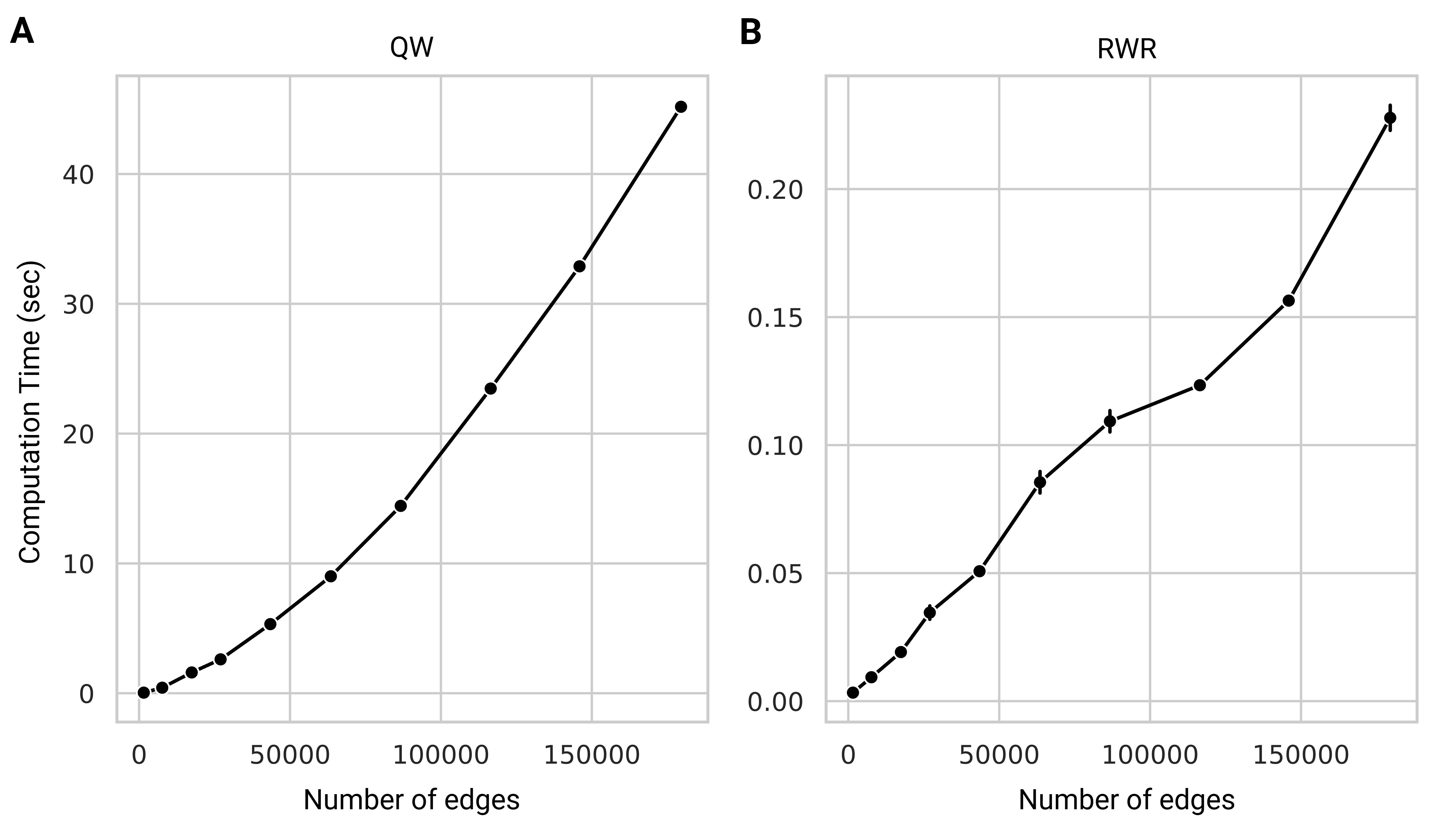
**

**Supplementary Figure 5.** Analysis of computational scalability for QW and RWR with respect to network size. The wall-clock time required for computation was measured across LCP subnetworks of varying sizes (represented by the number of edges). Data points represent the mean computation time over 10 independent trials, with error bars indicating the standard deviation. (A) Computation time for the QW algorithm. The runtime increases non-linearly with the number of edges, reflecting the higher computational cost associated with operations in the arc-based Hilbert space. (B) Computation time for the RWR algorithm. The runtime exhibits linear scalability, consistent with the theoretical complexity of sparse matrix-vector multiplication.

**
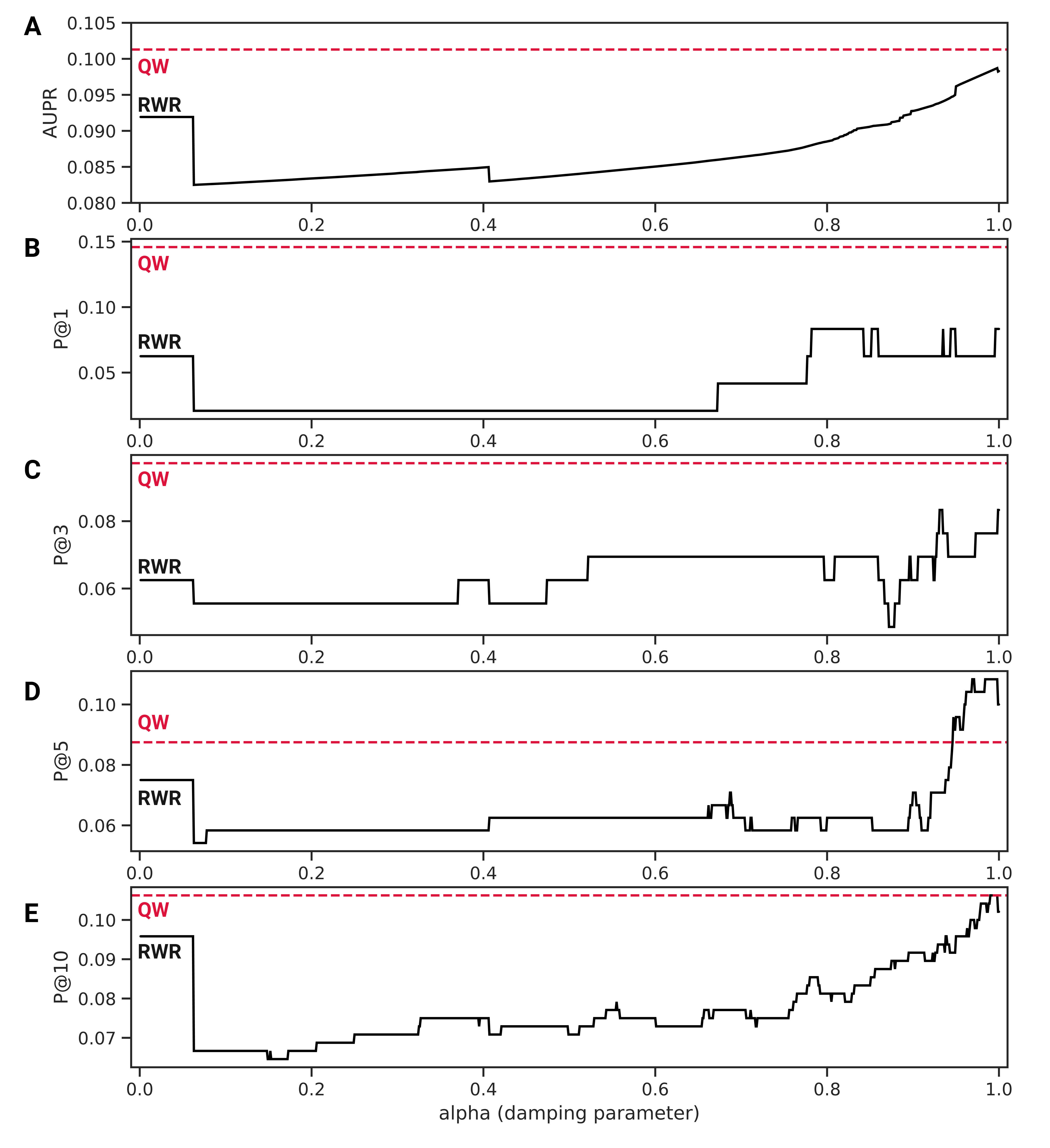
**

**Supplementary Figure 6.** Performance comparison between QW and RWR across the full range of the damping parameter. The RWR performance, calculated by varying the damping parameter alpha from 0 to 1 in increments of 0.01, is visualized as a solid black line. The red dashed line represents the performance of QW obtained using the optimal measurement mixing time (t=1212), serving as a constant reference for comparison. (A) Comparison of Area Under the Precision-Recall curve (AUPR). (B–E) Comparison of Precision at k (P@k) for the top 1%, 3%, 5%, and 10% of predictions, respectively. These plots demonstrate that QW generally outperforms or is comparable to the best-performing configuration of RWR, particularly in the top-ranked predictions (P@1% and P@3%).

**
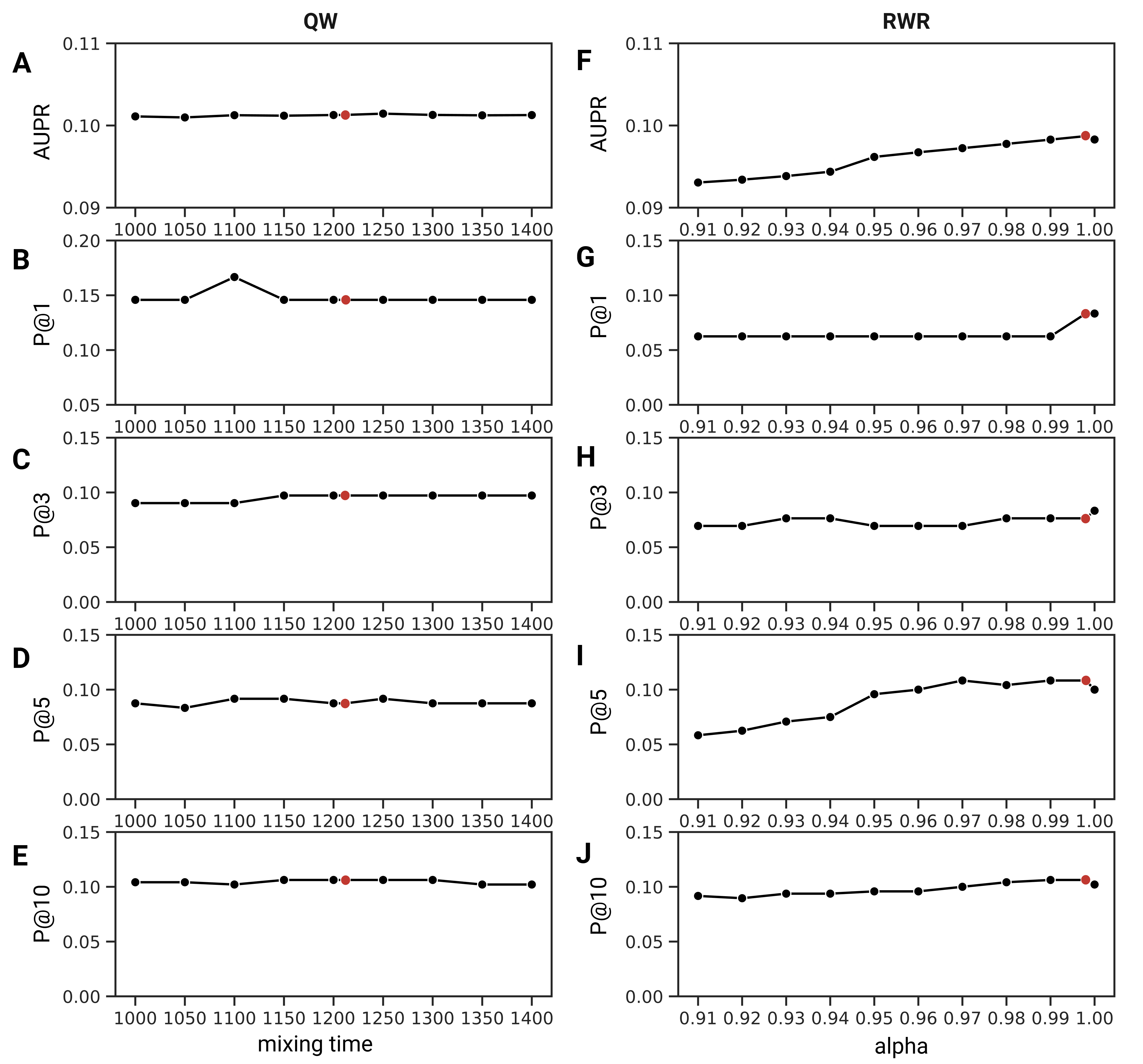
**

**Supplementary Figure 7. Robustness of LCP prediction performance with respect to hyperparameter variations.** The QW results are presented in the left column, while the RWR results are shown in the right column. The red dots indicate the performance obtained using the optimal hyperparameters selected for the main analysis (t=1212 for QW and alpha=0.998 for RWR). (A–E) Variations in AUPR and Precision at k% (P@1, P@3, P@5, and P@10) for QW, obtained by varying the measurement mixing time from 1000 to 1400 in increments of 50. (F–J) Corresponding performance metrics for RWR, obtained by varying the damping parameter alpha from 0.91 to 1 in increments of 0.01.

**
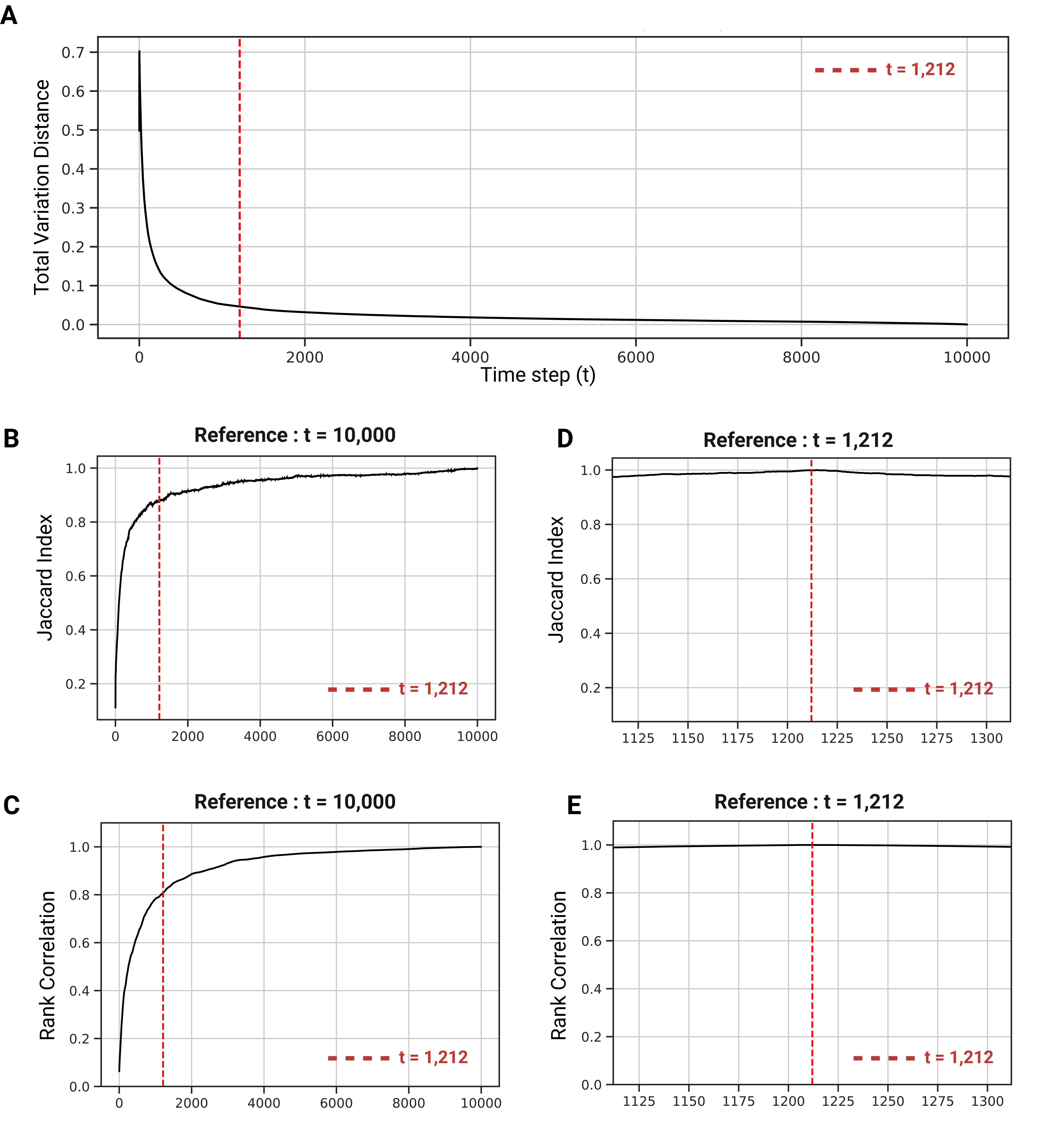
**

**Supplementary Figure 8. Convergence and stability analysis of the Quantum Walk algorithm.** (A) Total Variation Distance (TVD) calculated between the probability distribution at time t and the reference distribution at $t_{max}=10,000$. The red dashed line indicates the selected time point ($t=1,212$). (B, C) Similarity metrics for the top 947 prioritized proteins at time t compared to the reference distribution at $t_{max}=10,000$. The plots show the (B) Jaccard Similarity Index and (C) Spearman Rank Correlation Coefficient. (D, E) Local stability analysis comparing rankings within a window of $\pm100$ steps relative to the selected mixing time point ($t=1,212$). The plots display the (D) Jaccard Index and (E) Spearman Rank Correlation.

**Supplementary Table 1.** Intersecting protein lists between QW & RWR key proteins, and LCPs

| **QW Key Proteins &**  **Shallow LCP** | **RWR Key Proteins &**  **Shallow LCP** | **QW Key Proteins &**  **Deep LCP** | **RWR Key Proteins &**  **Deep LCP** |
| --- | --- | --- | --- |
| RALA | RAF1 | AGT | LCK |
| CTNNB1 | CTNNB1 | KNG1 | AGT |
| EGFR | EGFR | UNC5D | CXCL12 |
| GSK3B | CXCL8 | GRB7 | CD80 |
| APP | ICAM1 | IGF2R | CD86 |
| ITSN1 | KIT | CTSV | LEP |
| VWF | KDR | C3 | KNG1 |
| DSG1 | HGF | B4GALT2 | GFAP |
| STX1A | PECAM1 | B4GALT1 | C3 |
| FMR1 | HSP90B1 | FOLH1 | B4GALT2 |
| LUM | BCL2L1 | TG | B4GALT1 |
| GABARAPL1 | CREBBP | SFTPD | LRP5 |
| GDF15 | NPM1 | CPN1 |  |
| GHRL | NOTCH1 | F8 |  |
| NPY | GSK3B | GRID2 |  |
| FXN | PIK3CG | AKR1A1 |  |
| ATP1B2 | CLU | SCG5 |  |
| F7 | APP | RGMA |  |
| ESAM | MET | PANK1 |  |
| ACADM | GSN | NUDT9 |  |
| GABARAP | LAMP1 | NUDT15 |  |
| MFAP5 | ITSN1 | CST6 |  |
| NOVA1 | WDR5 | B3GAT3 |  |
| GAL3ST1 | AURKB | PTPRD |  |
|  | KAT5 | PDXK |  |
|  | LGALS3 | FCRL5 |  |
|  | MSH2 | LMAN2L |  |
|  | VWF |  |  |
|  | STX1A |  |  |
|  | FMR1 |  |  |
|  | APOH |  |  |
|  | BUD31 |  |  |
|  | SNRPG |  |  |
|  | POLR1C |  |  |
|  | RAD51 |  |  |
|  | STX6 |  |  |
|  | CHEK1 |  |  |
|  | GABARAPL1 |  |  |
|  | GHRL |  |  |
|  | NPY |  |  |
|  | RRM2 |  |  |
|  | COX5A |  |  |
|  | GABARAP |  |  |

**Supplementary Table 2.** Top 10 proteins of QW result

| **Name** | **QW rank** | **RWR rank** | **Cell location** | **QW probability**  **(Z-normalized)** | **RWR probability**  **(Z-normalized)** |
| --- | --- | --- | --- | --- | --- |
| ZNF114 | 2 | 838 | Nucleus | 0.003263 | 0.000213 |
| CLIP4 | 3 | 255 | Nucleus, Cell cortex | 0.002736 | 0.000491 |
| TRIM28 | 4 | 1064 | Nucleus | 0.001594 | 0.000183 |
| CISD3 | 5 | 261 | Mitochondrion | 0.001426 | 0.000487 |
| PPP2R2C | 6 | 1781 | Cytosol | 0.001140 | 0.000123 |
| FAU | 7 | 142 | Nucleus, Cytoplasm | 0.001065 | 0.000566 |
| CISD1 | 8 | 2083 | Mitochondrion outer membrane | 0.000969 | 0.000108 |
| TP53 | 9 | 2 | Nucleus, Cytoplasm, ER, ​ Mitochondrion matrix | 0.000911 | 0.001136 |
| CDC42 | 10 | 388 | Cell membrane, Cytoplasm | 0.000865 | 0.000405 |
| CISD2 | 11 | 1440 | Mitochondrion outer membrane, ER | 0.000832 | 0.000145 |

**Supplementary Table 3.** Top 20 GO ORA result of the CISDs related subnetwork

| **Ranking** | **GO id** | **Term description** | **Adjusted p-value** |
| --- | --- | --- | --- |
| 1 | GO:2000378 | negative regulation of  reactive oxygen species metabolic process | 3.48 x 10^-6^ |
| 2 | GO:0010506 | regulation of autophagy | 3.48 x 10^-6^ |
| 3 | GO:0000422 | autophagy of mitochondrion | 2.36 x 10^-5^ |
| 4 | GO:0061726 | mitochondrion disassembly | 2.36 x 10^-5^ |
| 5 | GO:0098780 | response to mitochondrial depolarisation | 2.36 x 10^-5^ |
| 6 | GO:1903008 | organelle disassembly | 8.50 x 10^-5^ |
| 7 | GO:2000377 | regulation of reactive oxygen species metabolic process | 8.50 x 10^-5^ |
| 8 | GO:0000423 | mitophagy | 1.01 x 10^-4^ |
| 9 | GO:2001242 | regulation of intrinsic apoptotic signaling pathway | 1.84 x 10^-4^ |
| 10 | GO:0072593 | reactive oxygen species metabolic process | 3.48 x 10^-4^ |
| 11 | GO:0070059 | intrinsic apoptotic signaling pathway  in response to endoplasmic reticulum stress | 3.48 x 10^-4^ |
| 12 | GO:0034976 | response to endoplasmic reticulum stress | 5.25 x 10^-4^ |
| 13 | GO:0098779 | positive regulation of mitophagy  in response to mitochondrial depolarization | 7.25 x 10^-4^ |
| 14 | GO:0061912 | selective autophagy | 8.60 x 10^-4^ |
| 15 | GO:0097193 | intrinsic apoptotic signaling pathway | 8.60 x 10^-4^ |
| 16 | GO:1904925 | positive regulation of autophagy of mitochondrion  in response to mitochondrial depolarization | 1.10 x 10^-3^ |
| 17 | GO:0036503 | ERAD pathway | 1.10 x 10^-3^ |
| 18 | GO:2001243 | negative regulation of intrinsic apoptotic signaling pathway | 1.10 x 10^-3^ |
| 19 | GO:1903599 | positive regulation of autophagy of mitochondrion | 1.10 x 10^-3^ |
| 20 | GO:1904923 | regulation of autophagy of mitochondrion  in response to mitochondrial depolarization | 1.10 x 10^-3^ |

**Supplementary Information A: Theoretical Background and Implementation of the Random Walk with Restart (RWR) Algorithm**

The Random Walk with Restart (RWR) algorithm is a network analysis method used to measure the global importance or proximity of nodes within a graph. It is a variant of the PageRank algorithm, originally developed for ranking web pages (Lawrence *et al.* 1999). RWR models a stochastic process that is periodically reset to a specific starting node or set of nodes, allowing for a personalized or query-dependent measure of importance.

Our analysis utilizes the PageRank algorithm as implemented in the Python library NetworkX (Hagberg et al. 2008), which provides a computationally efficient framework for this calculation. This appendix details the theoretical foundations of RWR and its practical implementation.

A simple random walk on a directed graph models a stochastic process where a "walker" traverses from its current node $u$ to an adjacent node $v$ by randomly selecting one of out-edges of u. The probability of transitioning from $u$ to $v$ is $1/L\left( u \right)$, where $L\left( u \right)$ is the out-degree of node $u$.

The importance or rank, $R\left( u \right)$ of a node $u$ can be defined as the stationary distribution of this Markov process. This represents the limiting probability that a walker will be at node $u$ after an infinite number of steps. This distribution is defined by the principle that a node's rank is derived from the rank of the nodes pointing to it:

$$R\left( u \right)= \sum_{v\in B_{u}} \frac{R\left( v \right)}{L\left( v \right)}$$

where $B_{u}$ is the set of nodes that point to $u$ (in-links). In matrix notation, this is expressed as $R = A^{T}R$, where $R$ is the vector of ranks and $A$ is the row-normalized adjacency matrix of the graph. The rank vector $R$ is thus the principal eigenvector of $A^{T}$.

The simple random walk model fails on graphs that are not strongly connected. Two primary issues arise. Rank sinks are groups of nodes that are connected in a cycle but have no out-edges leading outside the group. A walker entering this sink can never leave, and the group will eventually accumulate the entire rank of the graph, preventing convergence to a meaningful distribution. And dangling nodes are groups of nodes with no out-edges act as a sink, as a walker arriving there has nowhere to transition.

RWR solves these limitations by introducing a restart mechanism. The walker does not follow links indefinitely. At each step, it has two choices: With probability $\alpha$ (the damping parameter), it follows a random out-link. With probability $1-\alpha$, it restarts by jumping to any node in the graph, as defined by a personalization vector $P$. The vector $P$ (also known as the restart or personalization vector) is a probability distribution where $\sum P\left( u \right) = 1$.

This process is formalized by the following equation for a rank of node $u$ :

$$R\left( u \right)= \left( 1-\alpha\right)P\left( u \right)+ \alpha\sum_{v\in B_{u}} \frac{R\left( v \right)}{L\left( v \right)}$$

The first term represents the rank contributed by the restart. The second term represents the rank propagated through in-links.

In matrix notation, this is expressed as:

$$R=\left( 1-\alpha\right)P+\alpha A^{T}R$$

This formulation guarantees the ergodicity of the random walk process, thereby ensuring convergence to a unique stationary distribution, denoted as $R$. The semantic interpretation of this resulting vector depends on the configuration of the restart probability distribution. Specifically, in the context of Standard RWR, setting $P$ to a uniform distribution over all $N$ nodes (i.e., $P\left( u \right)=\frac{1}{N}$) allows the algorithm to compute the unbiased, global importance of each node based on the network topology. Conversely, in Personalized RWR (or Personalized PageRank), where $P$ is localized to a single seed node or a specific subset of nodes, the resulting vector $R$ quantifies the structural proximity or relevance of all other nodes with respect to the starting set.

**Implementation**

The rank vector $R$ is the solution to the linear system $R = \left( 1-\alpha\right)P + \alpha A^{T}R$. For large graphs, $R$ is found efficiently using the power iteration method. This method starts with an initial rank vector $R_{0}$and iteratively refines the estimate using the update rule:

$$R_{k+1}=\left( 1-\alpha\right)P+\alpha A^{T}R_{k}$$

This iteration is repeated until the change between $R_{k+1}$ and $R_{k}$ is negligible, typically measured by the $\left| R_{k+1} - R_{k} \right|$falling below a predefined strictly small tolerance $\varepsilon$ (e.g., ${1.0 x 10}^{-5}$).

**References**

Hagberg, Aric A., et al. "Exploring Network Structure, Dynamics, and Function Using NetworkX." Proceedings of the 7th Python in Science Conference, 2008, pp. 11–15.

Page, Lawrence, et al. “The PageRank citation ranking: Bringing order to the web”. Stanford infolab, 1999.

**Supplementary Information B: Theoretical Background and Implementation of Discrete-Time Quantum Walks (DTQW) Algorithm**

The Discrete-Time Coined Quantum Walk (DTQW) serves as a fundamental theoretical framework for analyzing quantum dynamics on graphs and advancing quantum information processing. As a quantum-mechanical generalization of the classical random walk, the DTQW replaces the stochastic evolution of its classical counterpart with a unitary evolution governed by the principles of superposition, interference, and entanglement (Venegas-Andraca 2012). These non-classical properties give rise to distinct dynamical behaviors, positioning the DTQW as a cornerstone for the development of novel quantum algorithms. This appendix details the mathematical formalism of the DTQW and outlines its implementation utilizing the Hiperwalk Python library (Motta *et al.* 2023).

The Discrete-Time Coined Quantum Walk (DTQW) operates within a composite quantum state space that encapsulates both the walker's position and its internal coin state. Unlike a classical random walk, which is defined solely on the vertex set of a graph, the coined quantum walk formalism necessitates tracking both the current location and the intended direction of movement. Consequently, the DTQW is defined on a Hilbert space $\mathcal{H}$ spanned by the directed edges, or arcs, of the graph. The computational basis of this space consists of vectors denoted as $\left| v,w \right\rangle$, where $v$ represents the current vertex (tail) and $w$ denotes an adjacent vertex (head). Physically, the basis state $\left| v,w \right\rangle$ signifies that the walker is localized at vertex $v$ with its internal coin state oriented towards $w$. For an undirected graph with $\left| E \right|$ edges, considering both directions for each edge results in a total state space dimension of $2\left| E \right|$. The total quantum state of the system at time $t$, denoted as $\left| \psi\left( t \right) \right\rangle$, is represented as a coherent quantum superposition of these $2\left| E \right|$ basis vectors.

The temporal dynamics of the quantum walk over a single discrete time step are governed by a global unitary operator, denoted as $U$. This operator is constructed via the sequential application of two distinct components: the Coin Operator ($C$) followed by the Shift Operator ($S$), such that:

$$U = S \cdot C$$

Consequently, the quantum state of the system after $t$ steps, $|\psi(t)\rangle$, is determined by the iterative application of $U$ to the initial state $|\psi(0)\rangle$:

$$\left| \psi\left( t \right) \right\rangle=U^{t}\left| \psi\left( 0 \right) \right\rangle$$

The Coin Operator ($C$) is responsible for mixing the probability amplitudes within the local subspace of each vertex, thereby creating the superposition that determines the walker's subsequent direction. Structurally, $C$ is represented as a block-diagonal matrix, where each block acts independently on the arcs originating from a specific vertex $v$. In this work, we employ the Grover coin, a widely adopted operator that performs an "inversion about the average." This operation uniformly mixes the amplitudes across all outgoing directions from a given vertex. For a basis state $|v, w\rangle$, where $v$ has degree $d\left( v \right)$, the Grover coin action is defined as:

$$C |v, w\rangle= \sum_{v' \in N(v)} (\frac{2}{d\left( v \right)} - \delta_{w, v'}) |v, v'\rangle$$

where $N\left( v \right)$ denotes the set of neighbors of $v$, and $\delta$ represents the Kronecker delta.

Following the coin operation, the Shift Operator ($S$) executes the physical displacement of the walker across the graph edges based on the updated amplitude distribution. We utilize the standard Flip-Flop shift operator, which maps a directed edge to its reversed counterpart. Formally, its action is defined as:

$$S |v, w\rangle= |w, v\rangle$$

This transformation signifies that the walker traverses the edge from $v$ to $w$. Upon completion of this step, the walker is localized at vertex $w$, with its internal state oriented back towards $v$ (the origin of the move), ready for the subsequent coin operation in the next time step.

After $t$ steps, the classical probability $p_{v}\left( t \right)$ of finding the walker at a specific vertex $v$ is calculated by summing the squared absolute values of the amplitudes for all arc states that have $v$ as their tail.

$$p_{v}\left( t \right)=\sum_{w\in N\left( v \right)} \left| \left\langle v,w | \psi\left( t \right) \right\rangle\right|^{2}$$

This probability distribution $p_{v}\left( t \right)$ is used to analyze the quantum walk's dynamics and evaluate the success probability of algorithms.

**Implementation**

The quantum system is initialized by defining the underlying graph structure via its adjacency matrix $A$. Consistent with the theoretical model, the evolution operator $U$ is constructed by composing the Grover coin operator and the Flip-Flop shift operator. The simulation begins by preparing a normalized initial state vector $|\psi(0)\rangle$ within the $2\left| E \right|$-dimensional Hilbert space. The walker is localized at a starting vertex with its coin state prepared in a specific superposition.

The temporal dynamics are simulated by iteratively applying the global unitary operator $U$ to the state vector. For a total duration $T$, the state at each time step $t$ is computed as $|\psi(t)\rangle= U |\psi(t-1)\rangle$. Finally, the quantum state at each step is mapped to a classical probability distribution. This is achieved by projecting the quantum state onto the position basis (summing the squared magnitudes of the amplitudes associated with all incoming arcs for each vertex) to obtain the vertex-level probability $P_{v}\left( t \right)$.

**References**

Motta, P., Bezerra, G. A., Santos, A. F., & Portugal, R. (2023, September). Hiperwalk: Simulation of Quantum Walks with Heterogeneous High-Performance Computing. In 2023 IEEE International Conference on Quantum Computing and Engineering (QCE) (Vol. 1, pp. 424-433). IEEE.https://doi.org/10.1109/QCE57702.2023.00055

Venegas-Andraca, S. E. (2012). Quantum walks: a comprehensive review. Quantum Information Processing, 11(5), 1015-1106.https://doi.org/10.1007/s11128-012-0432-5
